# Flexible Cu_2_ZnSn(S,Se)_4_ solar cells with over 10% efficiency and methods of enlarging the cell area

**DOI:** 10.1038/s41467-019-10890-x

**Published:** 2019-07-04

**Authors:** Kee-Jeong Yang, Sammi Kim, Se-Yun Kim, Kwangseok Ahn, Dae-Ho Son, Seung-Hyun Kim, Sang-Ju Lee, Young-Ill Kim, Si-Nae Park, Shi-Joon Sung, Dae-Hwan Kim, Temujin Enkhbat, JunHo Kim, Chan-Wook Jeon, Jin-Kyu Kang

**Affiliations:** 1 Convergence Research Center for Solar Energy, DGIST, Daegu, 42988 Korea; 2 Department of Physics, Incheon National University, Incheon, 22012 Korea; 3 School of Chemical Engineering, Yeungnam University, Gyeongsangbuk-do, 38541 Korea

**Keywords:** Solar cells, Solar cells

## Abstract

For kesterite copper zinc tin sulfide/selenide (CZTSSe) solar cells to enter the market, in addition to efficiency improvements, the technological capability to produce flexible and large-area modules with homogeneous properties is necessary. Here, we report a greater than 10% efficiency for a cell area of approximately 0.5 cm^2^ and a greater than 8% efficiency for a cell area larger than 2 cm^2^ of certified flexible CZTSSe solar cells. By designing a thin and multi-layered precursor structure, the formation of defects and defect clusters, particularly tin-related donor defects, is controlled, and the open circuit voltage value is enhanced. Using statistical analysis, we verify that the cell-to-cell and within-cell uniformity characteristics are improved. This study reports the highest efficiency so far for flexible CZTSSe solar cells with small and large areas. These results also present methods for improving the efficiency and enlarging the cell area.

## Abstract (web summary)

Flexibility and homogeneity are preferred properties for the kesterite solar modules to compete with silicon counterparts. Here, Yang et al. achieve these properties by designing a thin and multi-layered precursor structure and at the same time increase the open circuit voltage and device efficiency.

## Introduction

Considerable value has been attributed to renewable energy technology for preventing climate change, and solar photovoltaic (PV) devices are required to play a noteworthy role in carbon dioxide reduction^1,2^. To expand and disseminate PV technology, various applications, and low costs should be possible. Flexible solar cell technology enables applications such as building-integrated PV (BIPV) and mobile applications to be extended. In addition, low-cost flexible substrates can be used to lower manufacturing costs, which can contribute to the expansion of the renewable energy market by shortening the energy payback time. In addition to low-cost flexible substrates, the application of low-cost materials is required to realize low manufacturing costs. Kesterite materials (e.g., Cu_2_ZnSnS_4_ (CZTS), Cu_2_ZnSnSe_4_ (CZTSe), and Cu_2_ZnSn(S,Se)_4_ (CZTSSe)) are more beneficial for low-cost implementation than CdTe and Cu(In,Ga)Se_2_ (CIGS) solar cells^3^. However, CIGS solar cells exhibited a power conversion efficiency (*η*) of 22.8%^4^, while CZTSSe solar cells had a relatively low efficiency of 12.6%^5,6^. Flexible CIGS solar cell efficiencies were 20.4% on polyimide (PI) substrates^7^ and 17.7% on stainless steel (SS) foil substrates^8^. In contrast, flexible kesterite solar cell efficiencies were 7.04% on Mo foil substrates^9^ and 6.29% on SS foil substrates^10^. In addition, the CZTS submodule efficiency of a soda lime glass (SLG) substrate with a cell area larger than 1 cm^2^ was 11.3%^6^. For flexible kesterite solar cells, a low efficiency of 7.04% was observed only with a small cell area of ~0.53 cm^11^. High solar cell efficiencies are currently achievable with kesterite thin-film solar cells, as the phase instability of their quaternary and secondary phases and related defects can lead to electron-hole recombination and decrease the open circuit voltage (*V*_OC_) and current density (*J*_SC_) characteristics^2^. In CIGS thin-film solar cells, fewer secondary phases and defects form because of their superior phase stability, but in kesterite absorber layers, secondary phases and defects of various types can be produced^12^. Thus, the generation of secondary phases and defects must be controlled to limit the recombination loss. Moreover, to enter the market of flexible kesterite solar cells, it is necessary to advance both high-efficiency and large-area technology.

In this paper, we report methods of enhancing the efficiency and enlarging the cell area of flexible CZTSSe solar cells. For high-efficiency flexible kesterite solar cells, the control of the secondary phase and defect generation in the CZTSSe absorber layer is required^12–25^. Defect formation is related to the secondary phase^16,19^, and the defect density directly affects the open circuit voltage deficit (*E*_g_/*q* − *V*_OC_, where *E*_g_/*q* is the bandgap and *q* is the charge of an electron) characteristics^26–28^. As shown in Table 1, the *V*_OC_ deficit of the 7.04% efficiency flexible CZTSSe solar cell on a Mo foil substrate was 0.593 V^9^, whereas that of the 4.10% efficiency flexible CZTS solar cell on a SS430 substrate, which had the highest *V*_OC_ value of all flexible CZTS solar cells, was 0.638 V^29^. Therefore, the *V*_OC_ deficit improvement through defect formation control is very important to attain a high efficiency. The absorber layer of the flexible CZTSSe solar cell exhibited a higher efficiency^9,11^ than those of flexible CZTS^10,29–35^ and CZTSe solar cells^36–38^. In addition, cell-to-cell and within-cell uniformity characteristics must be secured to enlarge the cell area. With this goal, we report the device characteristics of 3- and 7-stacked precursor structures in small- and large-area cells.

**Table 1:** Summary of reported kesterite solar cells on flexible substrates and the highest efficiency CZTS-based solar cells on SLG substrates with small and large areas

Substrate	Absorber	Precursor deposition	Solar cell structure	*η* (%)	*V*_OC_ (V)	*J*_SC_ (mA cm^−2^)	FF(%)	Cell area (cm^2^)	Ref.
SLG	CZTSSe	Sputtering	SLG/Mo/**CZTSSe/**CdS/ZnO/ZnO:Al/MgF_2_	12.6	0.541	35.39	65.90	0.480	^6^
SLG	CZTSSe	Sputtering	SLG/Mo/**CZTSSe/**CdS/ZnO/ZnO:Al/MgF_2_	11.3	0.533	33.57	63.0	1.176	^6^
Mo foil	CZTSSe	Sputtering	Mo foil/**CZTSSe/**CdS/ZnO/ZnO:Al	7.04	0.498	30.06	47.02	0.530	^9^
Corning Willow glass	CZTSSe	CZTS nanoparticles ink	Willow Glas/Mo/**CZTSSe**/CdS/ZnO/ITO/Al-Ni grid	6.90	0.370	31.70	58.40	–	^29^
SS430	CZTS	Sputtering	SS430/Mo/**CZTS**/CdS/ZnO/ITO	6.29	0.628	17.40	57.51	0.24	^10^
SS430	CZTSe	Sputtering	SS430/Cr/Mo/i-ZnO/Mo:Na/Mo/**CZTSe**/CdS/ZnO/ZnO:Al	6.10	0.360	27.90	56.80	–	^36^
PI foil	CZTSe	Sputtering	PI/Mo/**CZTSe**/CdS/ZnO/ITO	4.90	0.337	27.20	53.20	–	^37^
SS430	CZTS	Sputtering	SS430/Mo/**CZTS**/CdS/ZnO/ITO	4.10	0.638	13.38	48.01	0.3–0.4	^28^
Mo foil	CZTS	Electro-deposition	Mo foil/Mo/**CZTS**/CdS/ZnO/ZnO:Al	3.82	0.473	18.80	42.90	0.35	^30^
SS	CZTSe	Sputtering	SS/Cr barrier/Mo/i-ZnO/**CZTSe**/CdS/ZnO/ZnO:Al	3.50	0.302	24.70	47.10	0.09	^38^
Corning Willow Glass	CZTS	Sputtering	Willow Glass/Mo/**CZTS**/CdS/ZnO/ITO	3.08	0.491	10.60	59.20	–	^31^
Mo foil	CZTS	Successive ionic layer adsorption and reaction	Mo foil/**CZTS**/CdS/i-ZnO/AZO/Ag	2.42	0.477	11.29	45.00	0.12	^32^
Mo foil	CZTS	Spin coating	Mo foil/**CZTS**/CdS/ZnO/AZO/Al	2.25	0.370	13.52	45.00	0.25	^33^
Al foil	CZTS	CZTS ink	Al foil/Mo/**CZTS**/ZnS/i-ZnO/ITO	1.94	0.484	8.90	45.10	–	^34^
Polyimide	CZTS	Screen printing	PI/Mo/**CZTS**/CdS/ZnO:Al	0.49	0.386	4.76	27.00	0.15	^35^

## Results

### Photovoltaic device properties with small cell areas

**Fig. 1:**
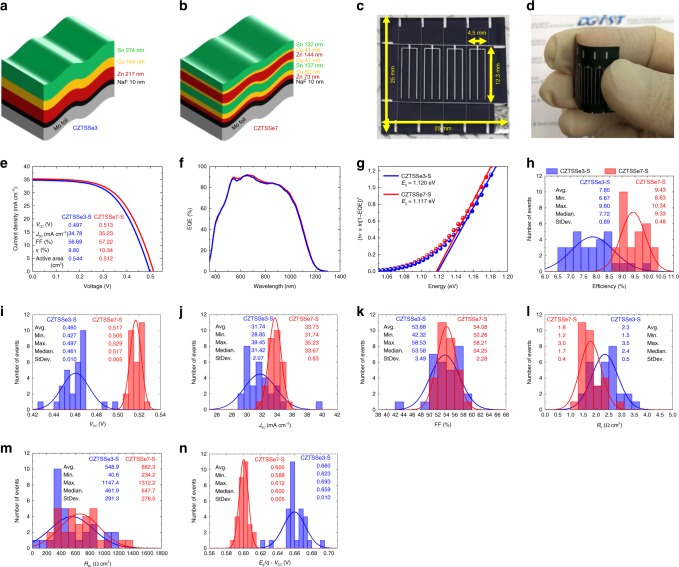
Photovoltaic device properties of flexible CZTSSe3-S and CZTSSe7-S. **a** 3-Stacked and **b** 7-stacked precursor structures. **c** Four cells ~4.5 mm × 12.3 mm in size are included in the Mo foil substrate, which is ~25 mm × 25 mm in size and **d** flexed sample image. All samples are with a small cell area of ~0.5 m^2^ for 30 devices of each type. **e** Current density-voltage curves, **f** EQE curves at a bias of 0 V, and **g** calculated bandgaps of CZTSSe3-S and CZTSSe7-S samples with the highest efficiencies (from KIER certificate, Supplementary Fig.1 and 2). **h** The average efficiency values are 7.85% with a StDev of 0.69 for CZTSSe3-S and 9.43% with a StDev of 0.48 for CZTSSe7-S. **i** The average *V*_OC_ values are 0.460 V with a StDev of 0.010 for CZTSSe3-S and 0.517 V with a StDev of 0.005 for CZTSSe7-S. **j** The average *J*_SC_ values are 31.74 mA cm^−2^ with a StDev of 2.07 for CZTSSe3-S and 33.73 mA cm^−2^ with a StDev of 0.83 for CZTSSe7-S. **k** The average FF values are 53.68% with a StDev of 3.49 for CZTSSe3-S and 54.08% with a StDev of 2.28 for CZTSSe7-S. **l** The average *R*_s_ values are 2.3 Ω cm^2^ with a StDev of 0.5 for CZTSSe3-S and 1.8 Ω cm^2^ with a StDev of 0.4 for CZTSSe7-S. **m** The average *R*_sh_ values are 548.9 Ω cm^2^ with a StDev of 291.3 for CZTSSe3-S and 662.3 Ω cm^2^ with a StDev of 276.0 for CZTSSe7-S. **n** The average *V*_OC_ deficit (*E*_g_*/q* − *V*_OC_) values are 0.660 V with a StDev of 0.010 for CZTSSe3-S and 0.600 V with a StDev of 0.005 for CZTSSe7-S

There were two types of designed precursor structures: one had a 3-stacked structure (Fig. 1a, CZTSSe3), and the other had a 7-stacked structure (Fig. 1b, CZTSSe7). As shown in Fig. 1c, four cells that were ~4.5 mm × 12.3 mm in size were included in the Mo foil substrate, which was ~25 mm × 25 mm in size. Figure 1d shows the flexed sample image. The devices were labeled CZTSSe3-S and CZTSSe7-S. Note that the ‘S’ in each sample label denotes a small cell area. Current-voltage characteristics were measured for 30 samples of CZTSSe3-S and CZTSSe7-S. Figure 1e–g shows the current density-voltage (*IV*) and external quantum efficiency (EQE) characteristics of the CZTSSe3-S and CZTSSe7-S samples with the highest efficiencies (Supplementary Figs. 1 and 2). It can be observed from Fig. 1e, g that the *V*_OC_ deficit of CZTSSe3-S was 0.623 V and that of CZTSSe7-S was 0.604 V. Figure 1f–l show the statistical current density-voltage characteristics of flexible CZTSSe3-S and CZTSSe7-S for 30 devices of each type. The efficiency (Fig. 1h), *V*_OC_ (Fig. 1i), *J*_SC_ (Fig. 1j), fill factor (FF) (Fig. 1k), *R*_s_ (Fig. 1l), and *R*_sh_ (Fig. 1m) values of CZTSSe7-S were better than those of CZTSSe3-S. In addition, the standard deviation (StDev) values of CZTSSe7-S were superior to those of CZTSSe3-S. Therefore, CZTSSe7-S devices exhibited cell-to-cell characteristics that were more uniform than those of CZTSSe3-S devices. In Fig. 1n, the *V*_OC_ deficit is expressed in terms of the relation between *E*_g_ and *V*_OC_, *E*_g_/*q* *−* *V*_OC_ ∝ *A* ln *N*, where *q* denotes the elementary charge, *A* is the diode ideality factor, and *N* is the concentration of recombination centers in the bulk CZTSSe absorber layer^26–28^. Defects in the absorber layer acted as electron-hole recombination centers^9^. From the *V*_OC_ deficit value (Fig. 1n), it was deduced that the defect-related *V*_OC_ loss was lower in the CZTSSe7-S absorber layer than that in the CZTSSe3-S absorber layer. Admittance spectroscopy (AS) was conducted to investigate the defect characteristics of the two types of precursor structures.

**Fig. 2:**
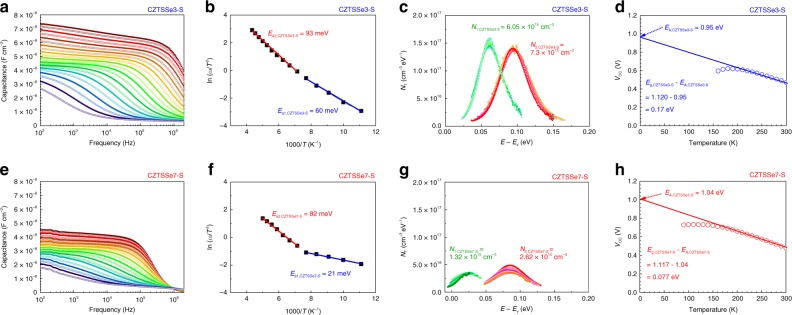
Defect activation energies of flexible CZTSSe3-S and CZTSe7-S. (Supplementary Figs. 3–6) **a**, **e** Admittance spectra measured in the temperature range of 90–300 K using probe frequencies from 20 Hz to 2 MHz. **b**, **f** Arrhenius plots of the inflection points of the capacitance function calculated from derivatives of the AS measurements. *E*_*a*_ denotes the main defect energy level within the absorber layer. **c**, **g** Concentrations of recombination centers (defect densities, *N*) derived from **a** and **e**. **d**, **h** Temperature dependence of *V*_OC_. The values of *E*_A_ for CZTSSe3-S and CZTSSe7-S are 0.95 and 1.04 eV, respectively. The values of *E*_g_ − *E*_A_ for CZTSSe3-S and CZTSSe7-S are 0.17 and 0.077 eV, respectively

### Defect analysis in CZTSSe absorber layers

AS measurements were used to analyze the energy levels of the defects inside the bandgaps of the CZTSSe absorber layer following previously reported procedures^16,17,19,20,26,27^. Figure 2a and e shows the AS measurement results of flexible CZTSSe solar cells with two types of precursor structures. To determine the defect activation energy, we constructed Arrhenius plots of the AS curve inflection points. Each Arrhenius plot was calculated via *ω*_0_ = 2*πν*_0_*T*^2^ exp(−*E*_a_*/kT*), where *ω*_0_ is the inflection point of the capacitance function, *E*_a_ is the energy level of the defect relative to the valence band maximum (*E*_*v*_), and *ν*_0_ is the pre-exponential factor^26^. The defects included vacancies (V_A_), interstitials (A_i_), A_B_ antisites (A replacing B), and defect clusters compensating for and attracting each other^12^. For CZTSSe3-S, as shown in Fig. 2b, the shallower main defect energy level was 60 meV (*E*_a1,CZTSSe3-S_), corresponding to the V_Cu_ defect, and the deeper main defect energy level was 93 meV (*E*_a2,CZTSSe3-S_), corresponding to Cu_Zn_ and Zn_Sn_ defects^12^. Furthermore, as shown in Fig. 2c, the range of energy levels was between 22 and 117 meV for the shallower defects and between 36 and 165 meV for the deeper defects. In the range of the shallower defect energy levels between 22 and 117 meV, the main defects, V_Cu_, and various other defects, including V_Zn_, Cu_Zn_, and Zn_Sn_ can be formed with defect densities of 6.05 × 10^15^ cm^−3^ (*N*_1,CZTSSe3-S_). Additionally, in the range of the deeper defect energy levels between 36 and 165 meV, the main defects, Cu_Zn_ and Zn_Sn_, and various other defects, including V_Cu_, V_Zn_, V_Sn_, and Cu_Sn_, can be formed with defect densities of 7.23 × 10^15^ cm^−3^ (*N*_2,CZTSSe3-S_). For CZTSSe7-S, as shown in Fig. 2f, the shallower main defect energy level was 21 meV (*E*_a1,CZTSSe7-S_), corresponding to the V_Cu_ defect, and the deeper main defect energy level was 82 meV (*E*_a2,CZTSSe7-S_), which corresponded to Cu_Zn_ and Zn_Sn_ defects^12^. Furthermore, as shown in Fig. 2g, the range of energy levels was between *E*_*v*_ and 49 meV for the shallower defects and between 47 and 130 meV for the deeper defects. In the range of the shallower defect energy levels between *E*_*v*_ and 49 meV, the main defects, V_Cu_, can be formed with defect densities of 1.32 × 10^15^ cm^−3^ (*N*_1,CZTSSe7-S_). Additionally, in the range of the deeper defect energy levels between 47 and 130 meV, the main defects, Cu_Zn_ and Zn_Sn_, and various other defects, including V_Cu_ and V_Zn_, can be formed with defect densities of 2.62 × 10^15^ cm^−3^ (*N*_2,CZTSSe7-S_). Regarding these results, the deeper defects within the CZTSSe3-S and CZTSSe7-S absorber layers were similar, but the defect density was lower and the defect distribution narrower in CZTSSe7-S than in CZTSSe3-S. These defect characteristics within the CZTSSe7-S absorber layer reflect improvements in the *V*_OC_ and *V*_OC_ deficit values (Fig. 1i, n). The defect energy level was shallower, the defect density was lower, and the defect distribution was narrower for the shallower defects of CZTSSe7-S (Fig. 2f, g) than for CZTSSe3-S (Fig. 2b, c). As shown in Fig. 2d, h, the activation energy (*E*_A_) for interfacial recombination in the depletion region could be assessed by calculating the temperature dependence of *V*_OC_. The correlation between *V*_OC_ and *T* can be expressed as *V*_OC_ = *E*_A_/*q* *−* *AkT*/*q* × ln (*J*_00_/*J*_*L*_), where *A* is the diode ideality factor, *kT*/*q* is the thermal voltage, *J*_00_ is the reverse saturation current prefactor, and *J*_L_ is the photocurrent^39–43^. When data in the range of *V*_OC_ measurement temperatures (90 K < *T* < 300 K) were linearly extrapolated, it was observed that *E*_A_ was the value at *T* = 0 K. If *E*_A_ is close to *E*_g_, the principal recombination mechanism is the Shockley–Read-Hall (SRH) process in the absorber depletion region^39,44^, whereas if recombination occurs in that region, the difference *E*_g_ − *E*_A_ increases. The differences in *E*_g_ − *E*_A_ for CZTSSe3-S (Fig. 2d) and CZTSSe7-S (Fig. 2h) were 0.17 and 0.077 eV, respectively. These results indicate that the density of recombination centers in the depletion region was lower for CZTSSe7-S than for CZTSSe3-S. Within the absorber layer, the types of main defects were similar, but the defect density of CZTSSe7-S was lower than that of CZTSe3-S. Additionally, recombination occurs more often in the absorber depletion region of CZTSSe-3S than in that of CZTSSe7-S. These characteristics resulted in improvements in the *V*_OC_ and *V*_OC_ deficit values (Fig. 1i, n). The reason that the defect characteristics differed between samples was that chalcogenization reaction mechanisms differed according to the stacked precursor structures^9,45–53^. As a result, the elemental ratio in the absorber layer was different, as summarized in Table 2. In the case of CZTSSe3, the variation in the Cu, Zn, and Sn content after the chalcogenization process was larger than that in the case of CZTSSe7. The more stable chalcogenization process was possible with the stacked precursor structure of CZTSSe7.

**Table 2:** Element ratios in the CZTSSe absorber layers for flexible CZTSSe3 and CZTSSe7 solar cells determined using an inductively coupled plasma (ICP) emission spectrometer

Sample		ICP data (mg kg^−1^)					Atomic ratios			
		Cu	Zn	Sn	S	Se	Cu/(Zn + Sn)	Zn/Sn	(S + Se)/(Cu + Zn;+ Sn)	S/(S + Se)
CZTSSe3	Precursor	1432	1300	1625	–	–	0.672	1.453	–	–
	Absorber	1172	1083	1371	978	34679	0.656	1.434	10.088	0.065
	absorber–precursor	−260	−217	−253	–	–	−0.015	−0.019	–	–
	(absorber–precursor) %	−18.17%	−16.70%	−15.59%	–	–	−2.29%	−1.32%	–	–
CZTSSe7	Precursor	1421	1356	1723	–	–	0.635	1.429	–	–
	Absorber	1329	1271	1622	982	36971	0.632	1.423	9.234	0.061
	absorber–precursor	−92	−85	−101	–	–	−0.003	−0.006	–	–
	(absorber–precursor) %	−6.50%	−6.28%	−5.86%	–	–	−0.42%	−0.45%	–	–

### Photovoltaic device properties with large cell areas

**Fig. 3:**
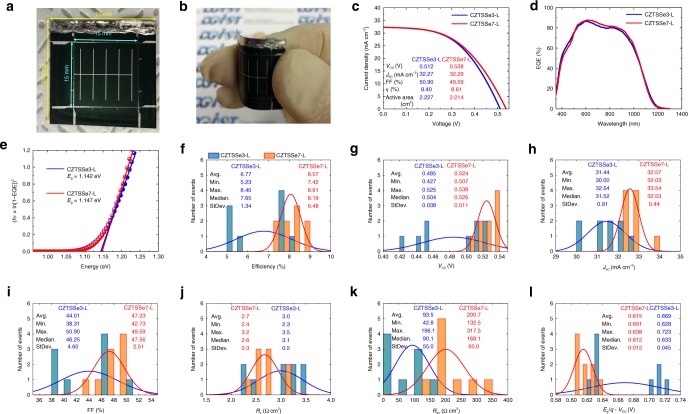
Photovoltaic device properties of flexible CZTSSe3-L and CZTSSe7-L. **a** 1 cell with a size of ~15 mm × 15 mm is included in the Mo foil substrate with a size of ~25 mm × 25 mm and **b** flexed sample image. All samples are with cell areas larger than 2 cm^2^ for 9 devices of each type. **c** Current density-voltage curves, **d** EQE curves at a bias of 0 V, and **e** calculated bandgaps of CZTSSe3-L and CZTSSe7-L samples with the highest efficiencies (from KIER certificate, Supplementary Figs. 7g, h, and 8m, n). **f** The average efficiency values are 6.77% with a StDev of 1.34 for CZTSSe3-L and 8.07% with a StDev of 0.48 for CZTSSe7-L. **g** The average *V*_OC_ values are 0.485 V with a StDev of 0.038 for CZTSSe3-L and 0.524 V with a StDev of 0.011 for CZTSSe7-L. **h** The average *J*_SC_ values are 31.44 mA cm^−2^ with a StDev of 0.91 for CZTSSe3-L and 32.57 mA cm^−2^ with a StDev of 0.44 for CZTSSe7-L. **i** The average FF values are 44.01% with a StDev of 4.60 for CZTSSe3-L and 47.23% with a StDev of 2.51 for CZTSSe7-L. **j** The average *R*_s_ values are 3.0 Ω cm^2^ with a StDev of 0.5 for CZTSSe3-L and 2.7 Ω cm^2^ with a StDev of 0.3 for CZTSSe7-L. **k** The average *R*_sh_ values are 93.5 Ω cm^2^ with a StDev of 55.0 for CZTSSe3-L and 200.7 Ω cm^2^ with a StDev of 60.0 for CZTSSe7-L. **l** The average *V*_OC_ deficit (*E*_g_*/q* *−* *V*_OC_) values are 0.669 V with a StDev of 0.045 for CZTSSe3-L and 0.615 V with a StDev of 0.012 for CZTSSe7-L

As shown in Fig. 3a, applying the precursor structure depicted in Fig. 1a, b, a 15 mm × 15 mm cell was fabricated on a 25 mm × 25 mm sized Mo foil substrate. The devices were labeled CZTSSe3-L and CZTSSe7-L. Note that the ‘L’ in each sample label denotes a large cell area. Figure 3b shows the flexed sample image. The current density-voltage characteristics were determined and certified for 9 samples each of CZTSSe3-L and CZTSSe7-L (Supplementary Figs. 7 and 8). Figure 3c–e shows the *IV* and EQE characteristics of the CZTSSe3-L and CZTSSe7-L samples with the highest efficiency values (Supplementary Figs. 7g and h, 8m and n). From Fig. 3c and e, it can be observed that the *V*_OC_ deficit of CZTSSe3-L was 0.512 V and that of CZTSSe7-L was 0.538 V. Figure 3f–l shows the statistical current density-voltage characteristics of CZTSSe3-L and CZTSSe7-L for 9 devices of each type. Similar to the small area samples in Fig. 1, the efficiency (Fig. 3f), *V*_OC_ (Fig. 3g), *J*_SC_ (Fig. 3h), FF (Fig. 3i), *R*_s_ (Fig. 3j), *R*_sh_ (Fig. 3k), and *E*_g_/*q* *−* *V*_OC_ (Fig. 3l) values of CZTSSe7-L were better than those of CZTSSe3-L. Additionally, the StDev values of CZTSSe7-L, except for those of *R*_sh_ (Fig. 3k), were superior to those of CZTSSe3-L. Therefore, in solar cells with areas larger than 2 cm^2^, CZTSSe7-L devices exhibited cell-to-cell characteristics that had a higher degree of uniformity than those of CZTSSe3-L devices.

**Fig. 4:**
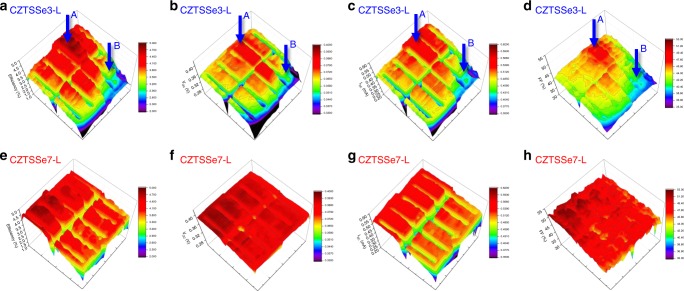
Within-cell uniformity of photovoltaic device properties by micro current-voltage (MIV). **a** Efficiency, **b** *V*_OC_, **c** *I*_SC_, and **d** FF of CZTSSe3-L with a 2.242 cm^2^ designated cell area (Supplementary Fig. 7m). **e** Efficiency, **f** *V*_OC_, **g** *I*_SC_, and **h** FF of CZTSSe7-L with a 2.214 cm^2^ designated cell area (Supplementary Fig. 8m)

To obtain large-area solar cells, it is important to improve not only the cell-to-cell uniformity but also the within-cell uniformity. To verify the degree of within-cell uniformity with two precursor structure types, micro current-voltage (MIV) analysis was performed. As shown in Fig. 4, the within-cell uniformity of CZTSSe7-L (Fig. 4e–h, Supplementary Fig. 8m) was better than that of CZTSSe3-L (Fig. 4a–d, Supplementary Fig. 7m). As shown for CZTSSe3-L in Fig. 4a–d, the characteristics at point A included a higher efficiency (Fig. 4a), *V*_OC_ (Fig. 4b), *I*_SC_ (Fig. 4c), and FF (Fig. 4d) than those at point B.

**Fig. 5:**
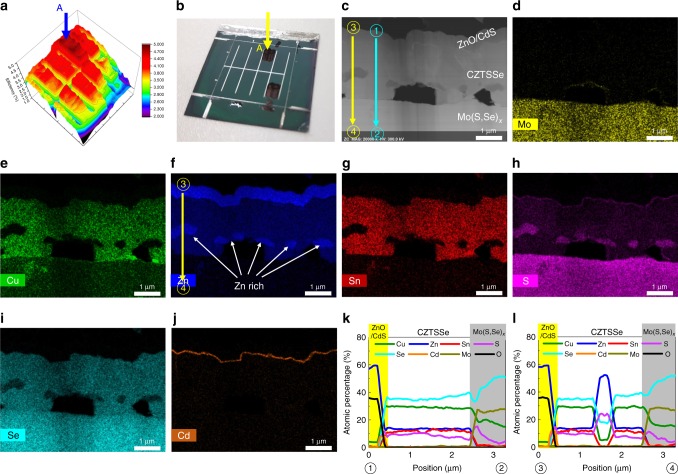
STEM-EDS mapping images of point A of flexible CZTSSe3-L. **a** Efficiency mapping image using MIV measurements. **b** Real sample image. **c** Cross-sectional STEM image. EDS mapping images of the CZTSSe3-L absorber layer showing mappings of **d** Mo, **e** Cu, **f** Zn, **g** Sn, **h** S, **i** Se, and **j** Cd. The elemental variations across the vertical and lateral directions in STEM images (**c**) were measured using EDS line scans. **k** EDS line scan of ①→②. **l** EDS line scan of ③→④

**Fig. 6:**
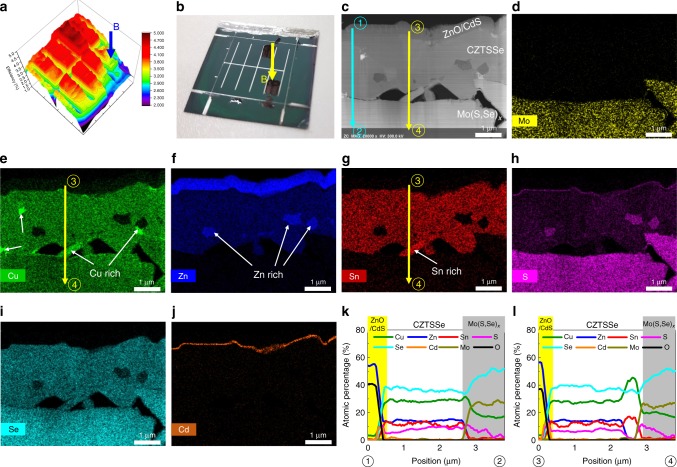
STEM-EDS mapping images of point B of flexible CZTSSe3-L. **a** Efficiency mapping image using MIV measurements. **b** Real sample image. **c** Cross-sectional STEM image. EDS mapping images of the CZTSSe3-L absorber layer showing mappings of **d** Mo, **e** Cu, **f** Zn, **g** Sn, **h** S, **i** Se, and **j** Cd. The elemental variations across vertical and lateral directions in the STEM images (**c**) were measured using EDS line scans. **k** EDS line scan of ①→②. **l** EDS line scan of ③→④

To investigate the differences between points A and B of the CZTSSe3-L device (Fig. 4a–d) in more detail, we performed scanning transmission electron microscopy-energy dispersive spectrometry (STEM-EDS) mapping. Figures 5 and 6 show the STEM-EDS mapping images of points A (Fig. 5a, b) and B (Fig. 6a, b) of CZTSSe3-L (Fig. 4a, d). As shown in Figs. 5c and  6c, voids were observed between CZTSSe and the Mo back contact by the Kirkendall effect^9,54^. The CdS buffer layers were well formed at points A and B of CZTSSe3-L (Figs. 5j and  6j). As shown in Figs. 5c, d, h, i, and  6c, d, h, i, the thickness of the Mo(S, Se)_x_ layer was very large because there was a very thick Mo foil substrate (0.1 mm). For point A of the CZTSSe3-L device (Fig. 5a, b), Cu (Fig. 5e) and Sn (Fig. 5g) were distributed uniformly. Therefore, the elemental distribution was uniform in the EDS line scan of ①→② (Fig. 5c and k). However, the EDS line scan of ③→④ (Fig. 5c, l) indicated that the elemental distribution fluctuated significantly in the Zn-rich region. In the Zn-rich region, the S content increased, and Cu, Sn, and Se contents decreased. Thus, zinc-sulfide/selenide secondary phase could have formed in this region. A zinc-sulfide/selenide secondary phase in the Zn-rich region was also observed at point B of the CZTSSe3-L device (Fig. 6f) and in the CZTSSe absorber layer according to our previously reported results^9,16,19,20^. As a result of the Zn-related secondary phase, various defects and defect clusters could be generated, which was a major factor in the deterioration of the CZTSSe solar cell efficiency^2,16^. Moreover, at point B of the CZTSSe3-L device (Fig. 6a, b), Cu- (Fig. 6e) and Sn-rich regions (Fig. 6g) were observed. The elemental distribution was uniform in the EDS line scan of ①→② at point B (Fig. 6c, k). However, the EDS line scan of ③→④ at point B (Fig. 6c, l) indicated that the elemental distribution fluctuated significantly in the Cu- and Sn-rich regions. In these regions, the Cu and Sn contents increased, and Zn and S contents decreased. Therefore, the Cu- and Sn-rich regions come out at similar positions, and copper-tin-selenide, copper-sulfide/selenide, and Sn-related secondary phases may also have been present^9^. Additionally, in these regions, Sn-related donor defects, such as Sn_Zn_ and Sn_Cu_, and defect clusters, such as 2Cu_Zn_ + Sn_Zn_, 2V_Cu_ + Sn_Zn_, and Cu_Sn_ + Sn_Cu_ could be formed in the CZTSSe absorber layer. These defects had a very notable defect energy level relative to the conduction band minimum (*E*_C_) and defect clusters caused the valence and conduction band shift. These defects and defect clusters resulted in a high characteristic loss, particularly with respect to *V*_OC_. Therefore, at point A of the CZTSSe3-L device (Fig. 5a, b), the defect situation may have been similar to CZTSSe3-S in Fig. 2. At point B of the CZTSSe3-L device (Fig. 6a, b), the defect situation may have been similar to CZTSSe7-S in Fig. 2, or there were many Sn-related defects and defect clusters. Compared with CZTSSe3, improvements in the cell-to-cell and within-cell uniformities and defect characteristics in CZTSSe7 were due to differences in the chalcogenization mechanism. Alloying the precursor structure as a stack of thick layers (CZTSSe3) could lead to the lateral separation of elements into steady-phase islands, producing a nonuniform element ratio at small length scales. However, the introduction of thin and multi-layered precursors (CZTSSe7) allowed uniform procedurals across the entire absorber layer. Additionally, as shown in Table 2, the change in composition before and after chalcogenization was also controllable in CZTSSe7, so the precursor design was advantageous not only for obtaining a high degree of efficiency and process reproducibility but also for fabricating large areas.

## Discussion

Through the application of a thin and multi-layered precursor structure, the efficiency of flexible CZTSSe solar cells and the possibility of large-area sizes were confirmed. The improvement in the cell-to-cell and within-cell uniformity characteristics is important for developing the efficiency and size of flexible CZTSSe solar cells. The most significant factor of these uniformities is the *V*_OC_ deficit loss due to the occurrence of defect and defect clusters. In particular, the control of deep-level defects, such as Sn-related donor defects, through control of the Sn-related secondary phase is important. Finally, chalcogenization processes are to be performed uniformly across the entire absorber layer to suppress the formation of defects and defect clusters and to minimize the *V*_OC_ deficit loss. As such, high efficiency and large-area technology by improving the cell-to-cell and within-cell uniformity will enable obtaining a high performance in other solar cell fields and contribute to the market expansion by increasing the productivity and application.

## Methods

### Solar cell fabrication

A 10-nm-thick NaF layer was deposited on the Mo foil substrate (0.1 mm thickness, MTI Co.) using a thermal evaporator for all samples. Metal precursors were deposited on the NaF layer using 99.99% pure Cu, Zn, and Sn sputtering targets with two different stacking orders. The layers were deposited under sputtering powers of 150 W, 300 W, and 300 W for the Cu, Zn, and Sn targets, respectively, at a working pressure of 1 mTorr in an Ar atmosphere. The following precursor stacking orders were employed: Mo foil/NaF (10 nm)/Zn (217 nm)/Cu (164 nm)/Sn (274 nm), which was labeled CZTSSe3; and Mo foil/NaF (10 nm)/Zn (73 nm)/Cu (82 nm)/Sn (137 nm)/Cu (41 nm)/Zn (144 nm)/Cu (41 nm)/Sn (137 nm), which labeled CZTSSe7. Note that the number in each precursor label denoted the number of precursor layers it contained. For the sulfo-selenization process, all samples were put in an annealing jig that consisted of a Se boat made of quartz, a sample holder made of SiC-coated graphite, and a quartz cover plate. Before the sulfo-selenization process was started, Se (250 mg), H_2_S (diluted with 250 sccm of 90 vol% Ar for 8 min) and Ar (2000 sccm, 8 min) gas were supplied to the rapid thermal processing (RTP) chamber. When the pressure reached 700 Torr, the supply of all gases was stopped and the heating process was performed. To avoid the decomposition of the CZTSSe, the sulfo-selenization process was performed using an RTP chamber at 700 Torr. The samples were heated from room temperature to 300 °C for 560 s and then maintained at 300 °C for 900 s. Subsequently, the sample was heated from 300 °C to 480 °C during 1800 s and then maintained at 480 °C for 600 s. A 50-nm-thick CdS buffer layer was deposited by CBD from a bath containing cadmium sulfate (CdSO_4_), ammonium hydroxide (NH_4_OH), thiourea (NH_2_CSNH_2_), and DI H_2_O. The bath solution was composed of 732 ml of DI H_2_O, 80 ml of NH_4_OH ammonium hydroxide, 100 ml of cadmium sulfate (0.015 M CdSO_4_), and 50 ml of thiourea (1.5 M NH_2_CSNH_2_). The pH range is commonly 11–11.5 in a thin-film deposition process. The solution is heated by bath with continuous stirring to the required temperature of deposition, and the temperature is controlled to within ± 1 °C at 65 °C. After being deposited for 13 min, the film was cleaned using ultrasonic in DI H_2_O to eliminate any adhered particles. Next, a 50-nm-thick intrinsic ZnO layer and a 300-nm-thick Al-doped ZnO (AZO) layer were deposited by RF sputtering. An intrinsic ZnO layer was deposited under sputtering power of 150 W at a working pressure of 6 mTorr for 750 s. An AZO layer was deposited under a sputtering power of 200 W at a working pressure of 2 mTorr at 200 °C for 3000 s. Finally, a 500-nm-thick Al collection grid was deposited on the top of the device using thermal evaporation.

### Solar cell characterization

The samples were characterized using a solar simulator, EQE analysis, ICP emission spectrometry, AS, MIV analysis, and STEM-EDS. The current-voltage characteristics were determined under a simulated air mass of 1.5 global (AM 1.5 G) spectrum and 100 mW cm^−2^ (1 sun) illumination at 25 °C using a 94022A solar simulator (Newport Co.) at the Daegu Gyeongbuk Institute of Science and Technology (DGIST), and a solar simulator (WACOM, WXS-155S-L2 (Class AAA)) at the Korea Institute of Energy Research (KIER) was used for certification. A class AAA solar simulator, which was described in IEC 60904-9, and a WPVS packaged calibrated reference solar cell as described in IEC 60904-2 were used. In addition, KIER certified the calibration of the reference solar cell. For the voltage scan, the average of 4 *IV* curves was obtained from forward and reverse voltage sweeps. Each *IV* parameter from the forward and reverse sweeps indicated a StDev within ~1%. There were ~200 data points per *IV* sweep. Samples were measured with a 2 ms sampling delay time for each data point measurement, *i*.e., ~1–2 s for 1 *IV* curve sweep, and approximately 30 s of light soaking and 15 s of dwell time between each *IV* curve sweep. The EQE values were obtained using a SOMA Optics (S-9230) at KIER. ICP (Shimadzu Co., model ICPS-8100) measurements were taken to analyze compositions of the absorber layers. AS measurements were performed to assess the defect energy levels inside the bandgaps of the CZTSSe absorber layer and within the temperature range of 90–300 using an E4980A LCR meter (Agilent Co.), which utilized probe frequencies from 20 Hz to 2 MHz. The measurements were conducted using a temperature tolerance of ± 0.05 K or less. To verify the defect activation energy, we calculated Arrhenius plots of the AS curve inflection points. MIV values were obtained by placing a device on a microscope stage and irradiating a red and blue laser with a 3 mW output power through a ×50 objective lens with an optical fiber guide. The size of the laser beam spot was ~100 μm in diameter. The measured device with dimensions of 15 mm × 15 mm was divided into four sections, and the MIV was measured at 20 × 20 points at intervals of 0.4 mm for each section. STEM-EDS (Bruker Co., model QUANTAX-200) measurements were performed to analyze the elements of the absorber layers and map out the elemental distribution.

## Supplementary information

Supplementary Information

Solar Cells Reporting Summary

## Data Availability

The data that support the findings of this study are available from the corresponding author (K.J. Yang, kjyang@dgist.ac.kr) upon reasonable request.
