# Supplementary material for: Flexible Cu2ZnSn(S,Se)4 solar cells with over 10% efficiency and methods of enlarging the cell area
Source: Nat Commun. 2019 Jul 4;10:2959. doi: 10.1038/s41467-019-10890-x (PMC6609618; doi:10.1038/s41467-019-10890-x)
Supplement: Supplementary file 1 — Supplementary Information [file 41467_2019_10890_MOESM1_ESM.docx]

**Supplementary Information**

**Flexible Cu_2_ZnSn(S,Se)_4_ solar cells with over 10% efficiency and methods of enlarging the cell area**

**Kee-Jeong Yang^1★^, Sammi Kim^1^, Se-Yun Kim^1^, Kwangseok Ahn^1^, Dae-Ho Son^1^, Seung-Hyun Kim^1^, Sang-Ju Lee^1^, Young-Ill Kim^1^, Si-Nae Park^1^, Shi-Joon Sung^1^, Dae-Hwan Kim^1^, Temujin Enkhbat^2^, JunHo Kim^2^, Chan-Wook Jeon^3^, Jin-Kyu Kang^1★^**

^1^Convergence Research Center for Solar Energy, DGIST, Daegu, Korea.

^2^Department of Physics, Incheon National University, Incheon, Korea.

^3^School of Chemical Engineering, Yeungnam University, Gyeongsangbuk-do, Korea.

^★^e-mail: kjyang@dgist.ac.kr; apollon@dgist.ac.kr


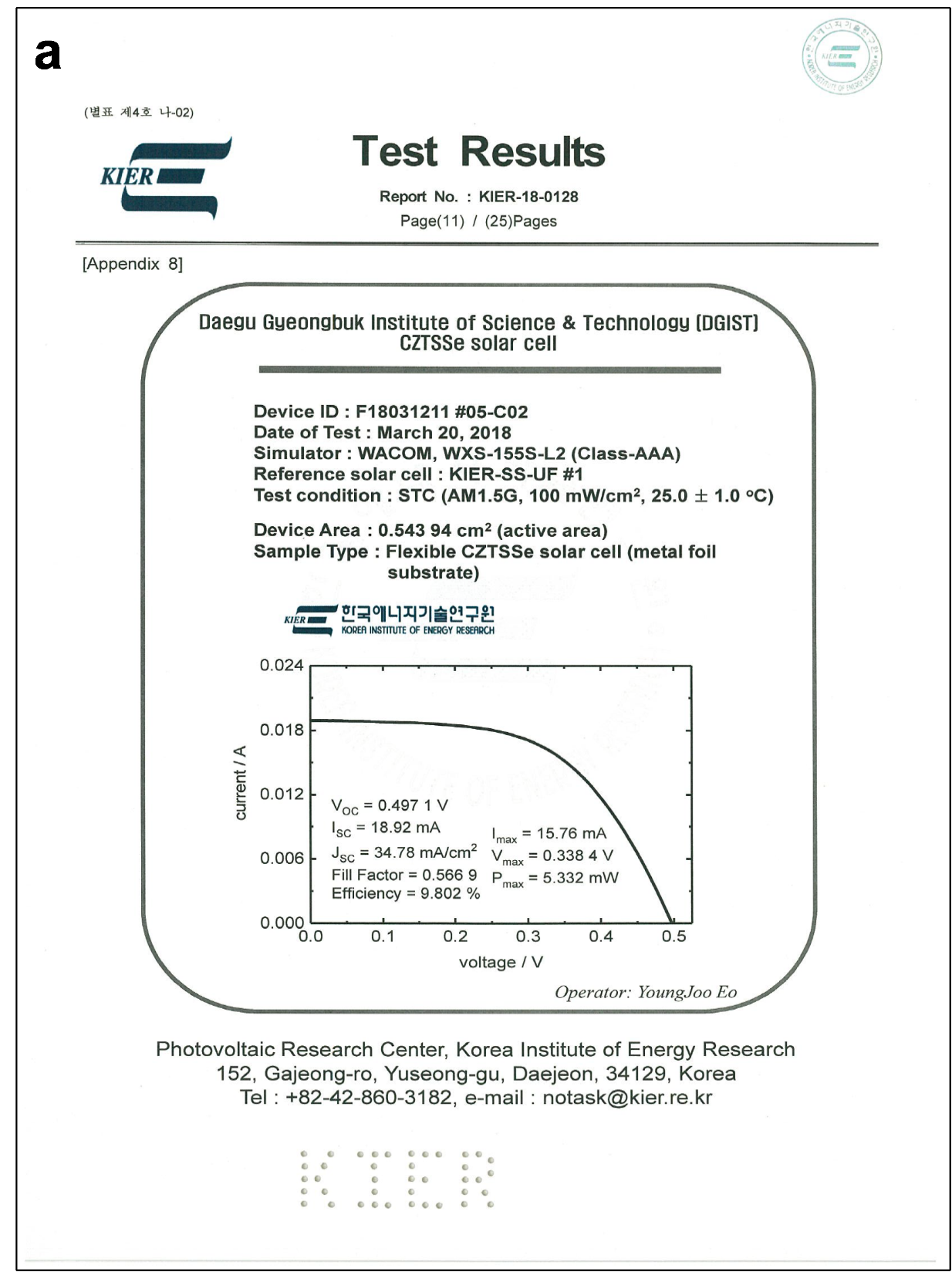


**Supplementary Figure. 1** The certificate of a 9.802% efficiency CZTSSe3-S flexible solar cell with an area of 0.544 cm^2^ (from KIER). **a** IV characteristics.


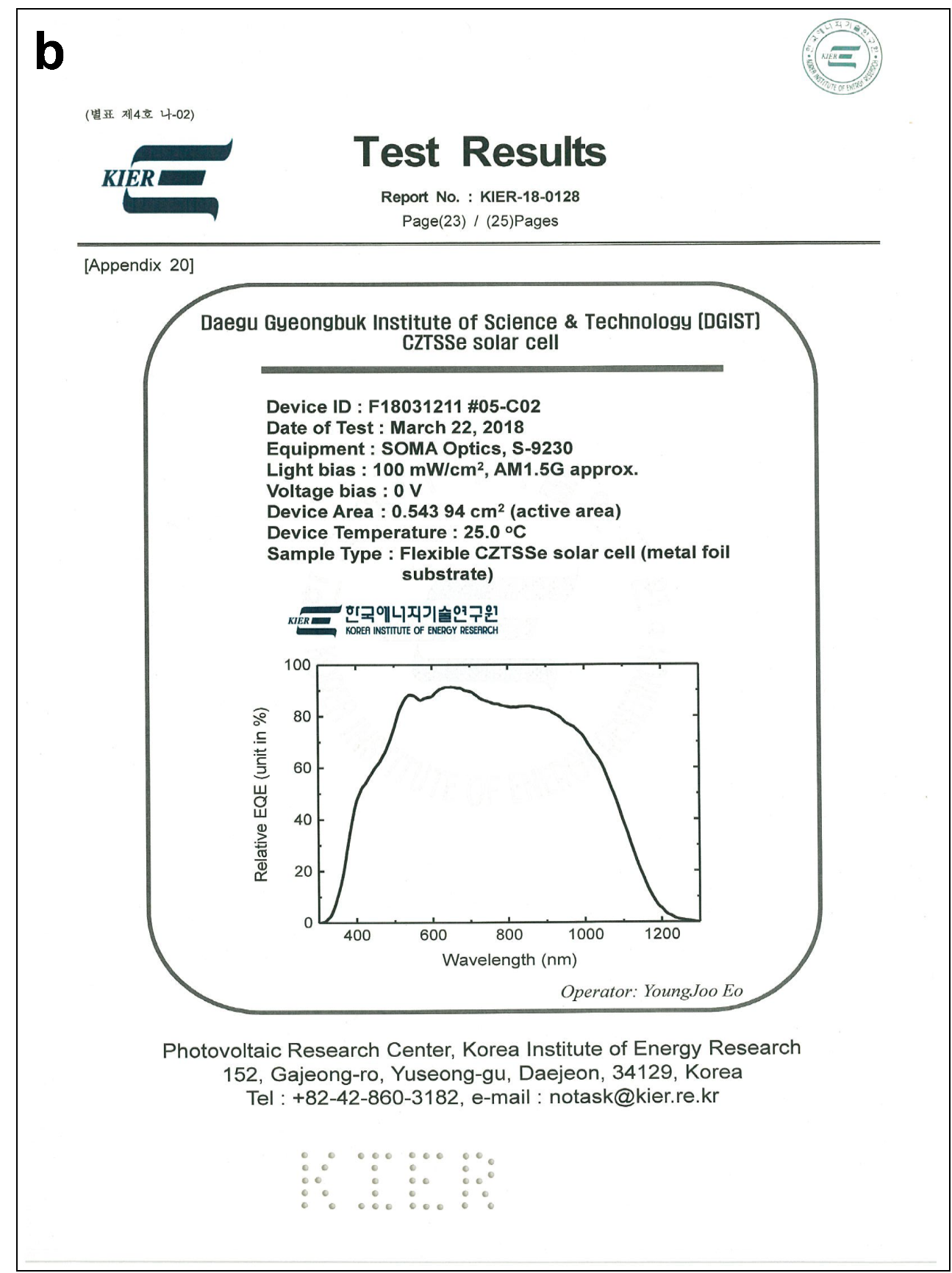


**Supplementary Figure. 1** *(continued)* **b** EQE characteristics.


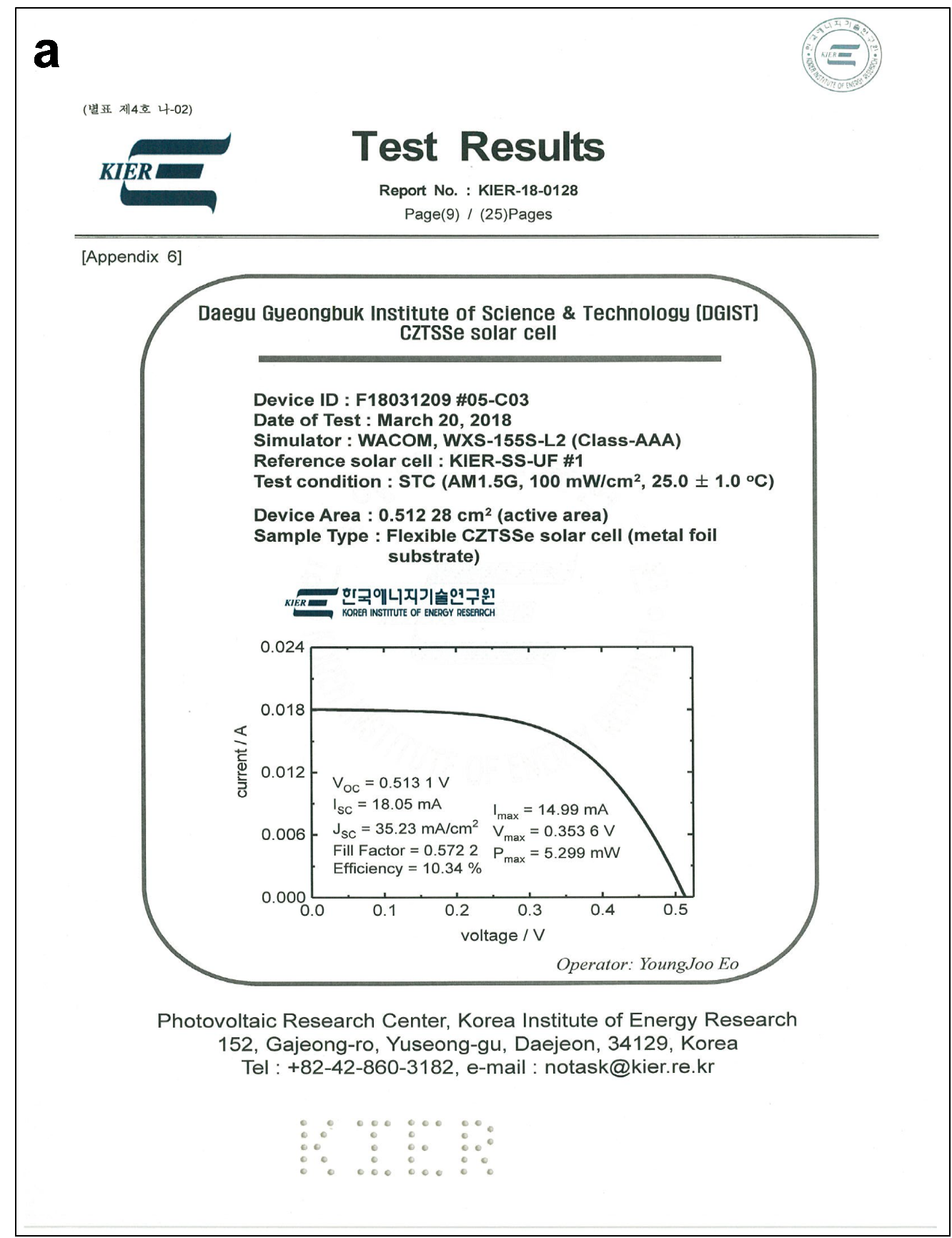


**Supplementary Figure. 2** The certificate of a 10.34% efficiency CZTSSe7-S flexible solar cell with an area of 0.512 cm^2^ (from KIER). **a** IV characteristics.


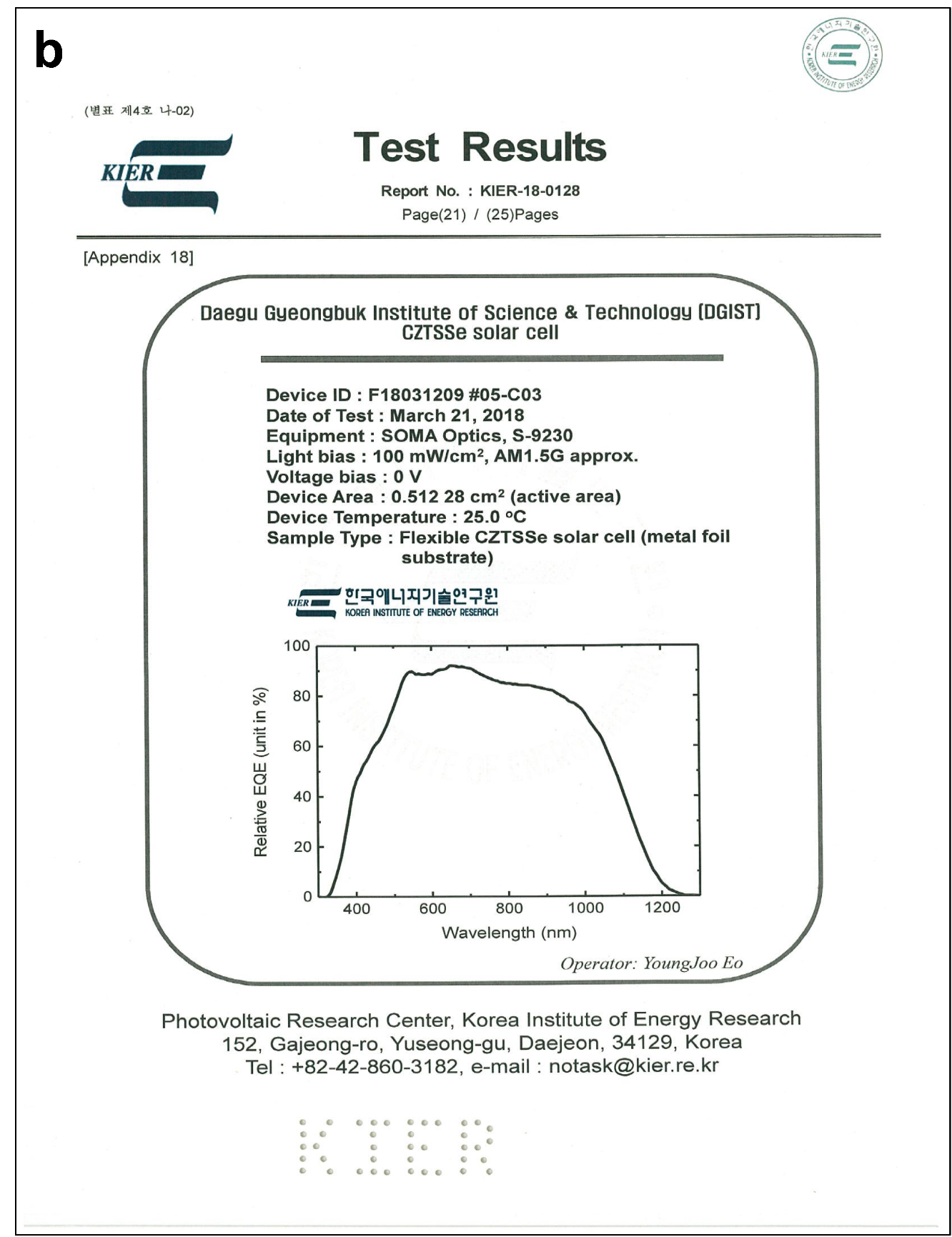


**Supplementary Figure. 2** *(continued)* **b** EQE characteristics.

**AS measurement analysis**

Defect distribution from the dependence of the capacitance on the angular frequency is determined as following equation^1^.

$\frac{dC}{d\omega}= \frac{dC}{dE_{\omega}} \frac{dE_{\omega}}{d\omega}$

$\frac{dE_{\omega}}{d\omega}= -\frac{kT}{\omega}$

Therefore, analogous peak plots can be determined from the AS data as using following derivative.

$\frac{dC}{dE_{\omega}}= -\frac{\omega}{kT}$ $\frac{dC}{d\omega}$


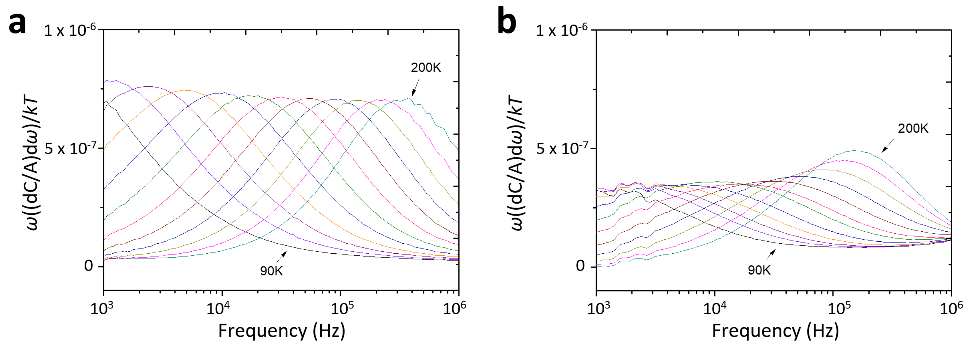


**Supplementary Figure. 3** Peak plots derived from AS data **a** CZTSSE3-S and **b** CZTSSe7-S.


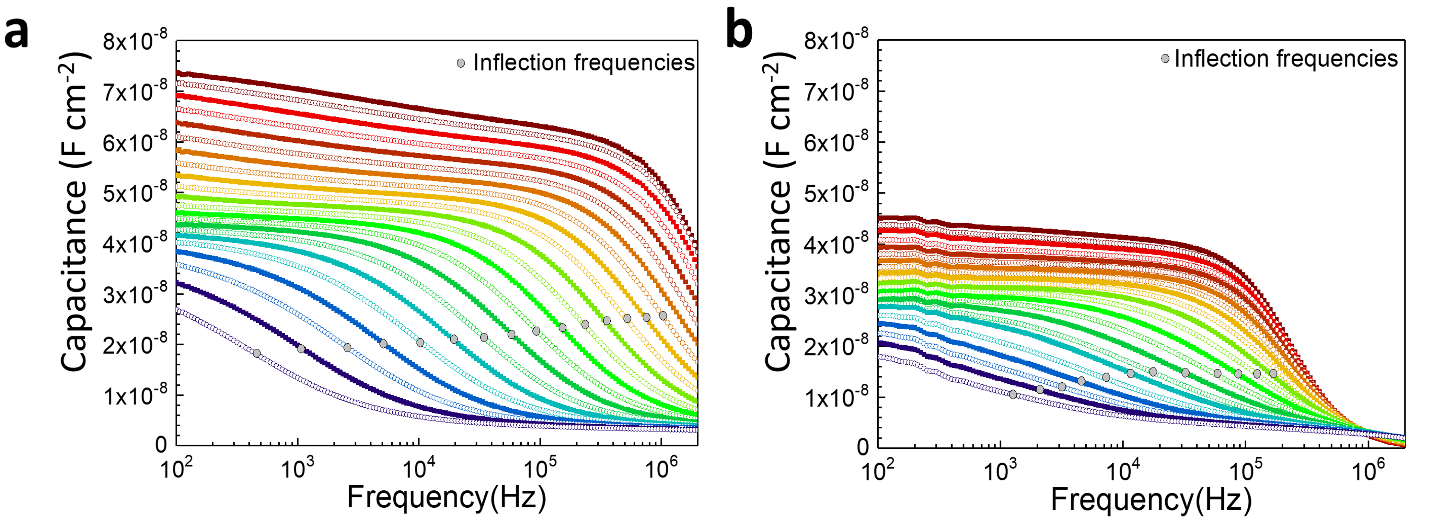


**Supplementary Figure. 4** AS inflection points of **a** CZTSSe3-S and **b** CZTSSe7-S.

The peak points and inflection points can be plotted as Arrhenius plot which is graphed as function of 1/*T* vs ln(*ω*/*T*^2^).

**
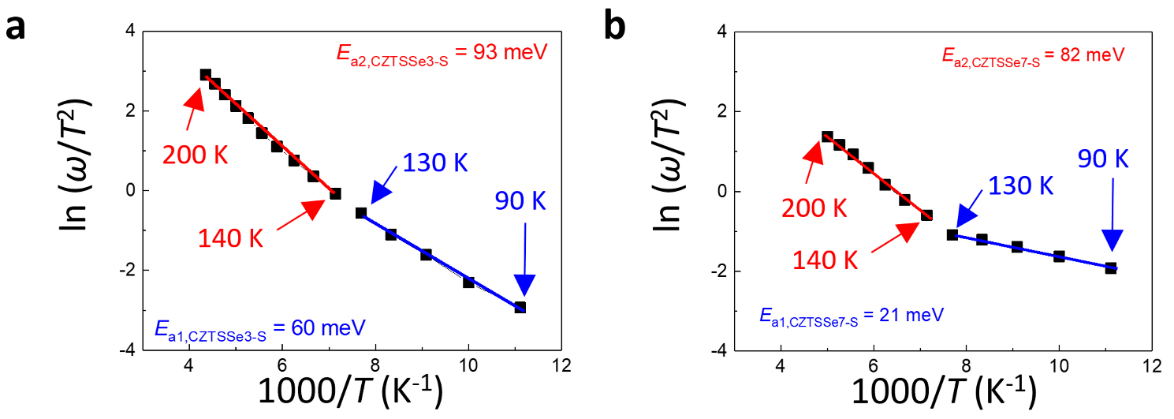
**

**Supplementary Figure. 5** Arrhenius plots of the inflection points of the capacitance function calculated from derivatives of the AS measurements. **a** CZTSSE3-S and **b** CZTSSe7-S.

Arrhenius plot slope and linear fit line yields the trap energy *E*_a_ and the intercept gives the value of apparent capture cross section $\sigma_{c}$ regarding to following equation^2^.

$e_{T}=N_{c,\nu}\sigma_{c}\nu_{th}exp(-\frac{E_{T}}{kT})$

The peak plots as shown in Supplementary Fig. 3 can be transformed into *N*_t_(*E_ω_*) vs *E_ω_* by using $\sigma_{c}$ to rescale *ω* axis to *E_ω_* and calculating *N*_t_(*E_ω_*) by following equation^1^.

$$N_{t}\left( E_{\omega} \right)= -\frac{2V_{bi}^{3/2}}{W\sqrt{e}\sqrt{eV_{bi}-\left( E_{g}-E_{\omega} \right)}}\frac{\omega}{kT}\frac{dC}{d\omega}$$

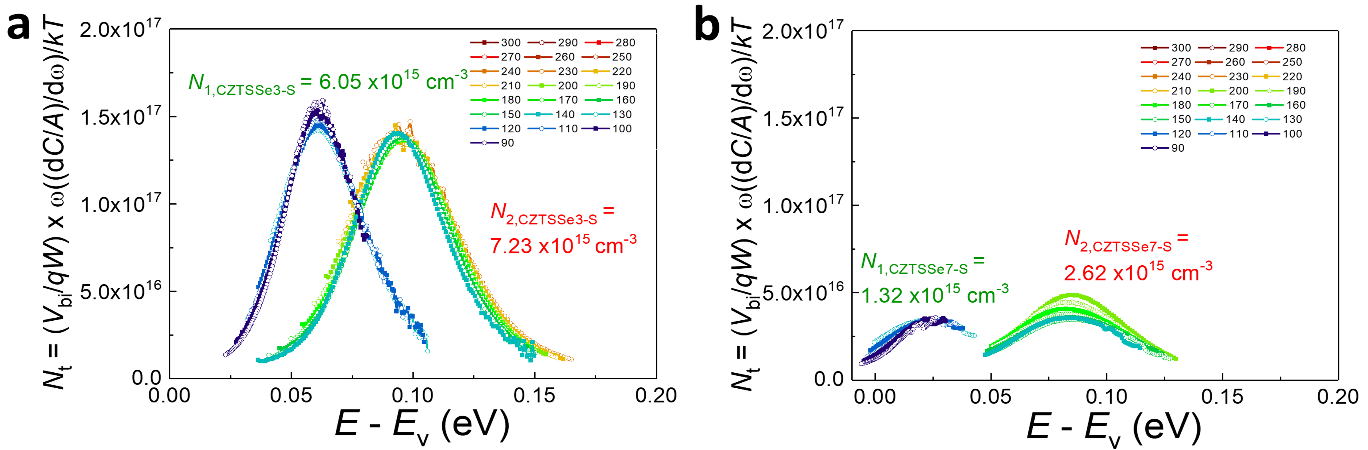


**Supplementary Figure. 6** Concentrations of defect densities derived from Supplementary Fig. 4. **a** CZTSSE3-S and **b** CZTSSe7-S.


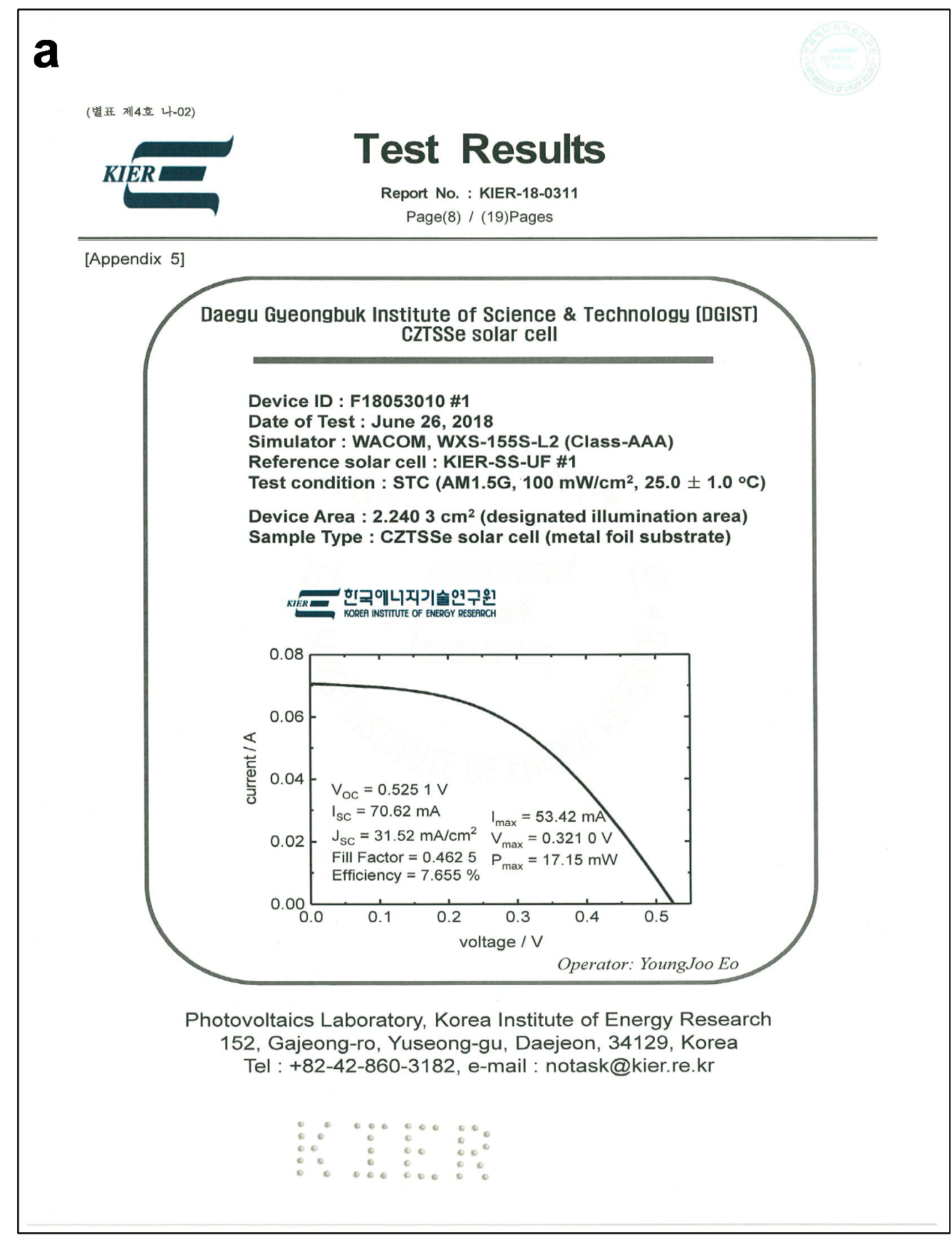


**Supplementary Figure. 7** The certificate of CZTSSe3-L flexible solar cells for 9 devices (from KIER). **a** IV characteristics of a 7.655% efficiency cell with an area of 2.240 cm^2^.

**
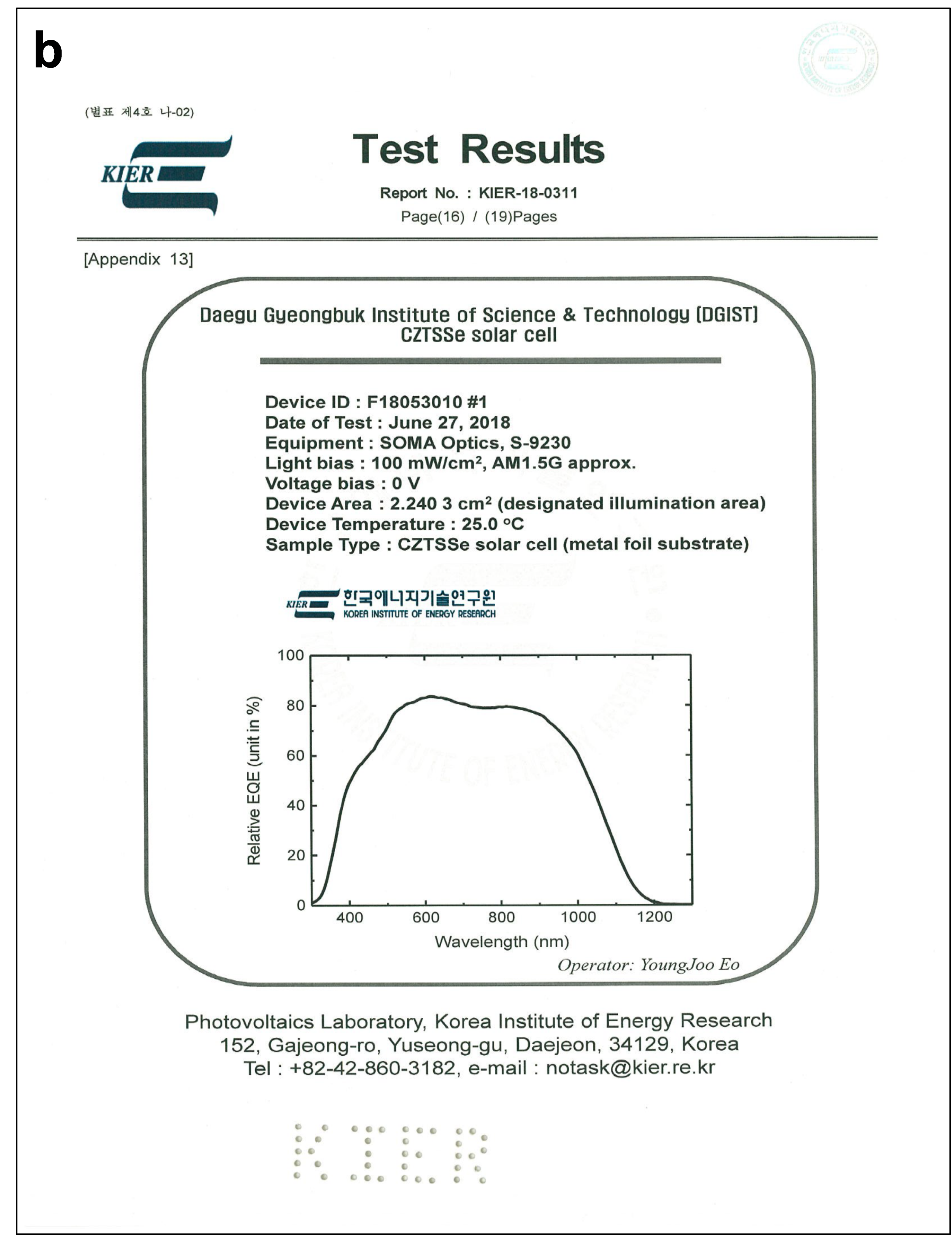
**

**Supplementary Figure. 7** *(continued)* **b** EQE characteristics of a 7.655% efficiency cell with an area of 2.240 cm^2^.

**
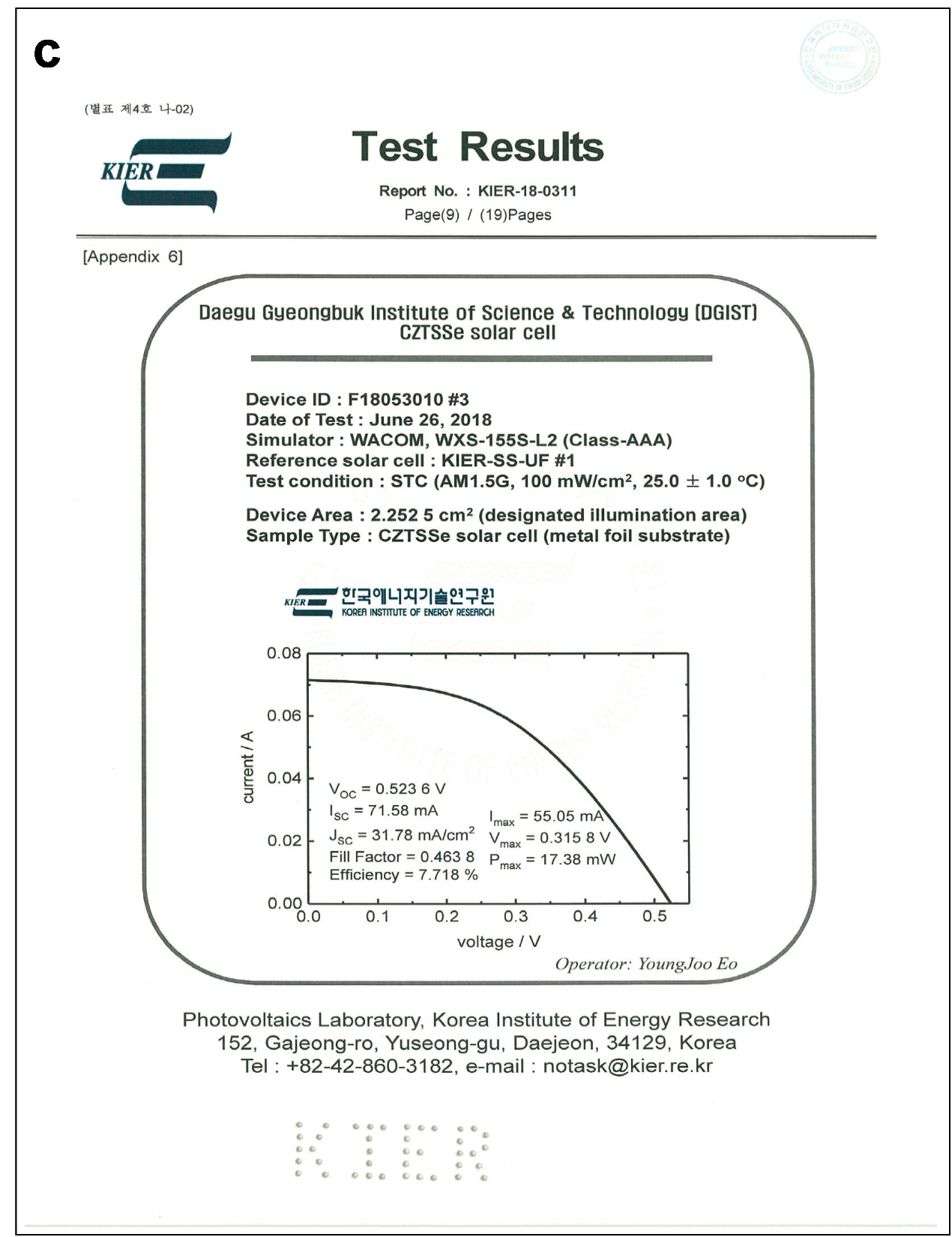
**

**Supplementary Figure. 7** *(continued)* **c** IV characteristics of a 7.718% efficiency cell with an area of 2.252 cm^2^.


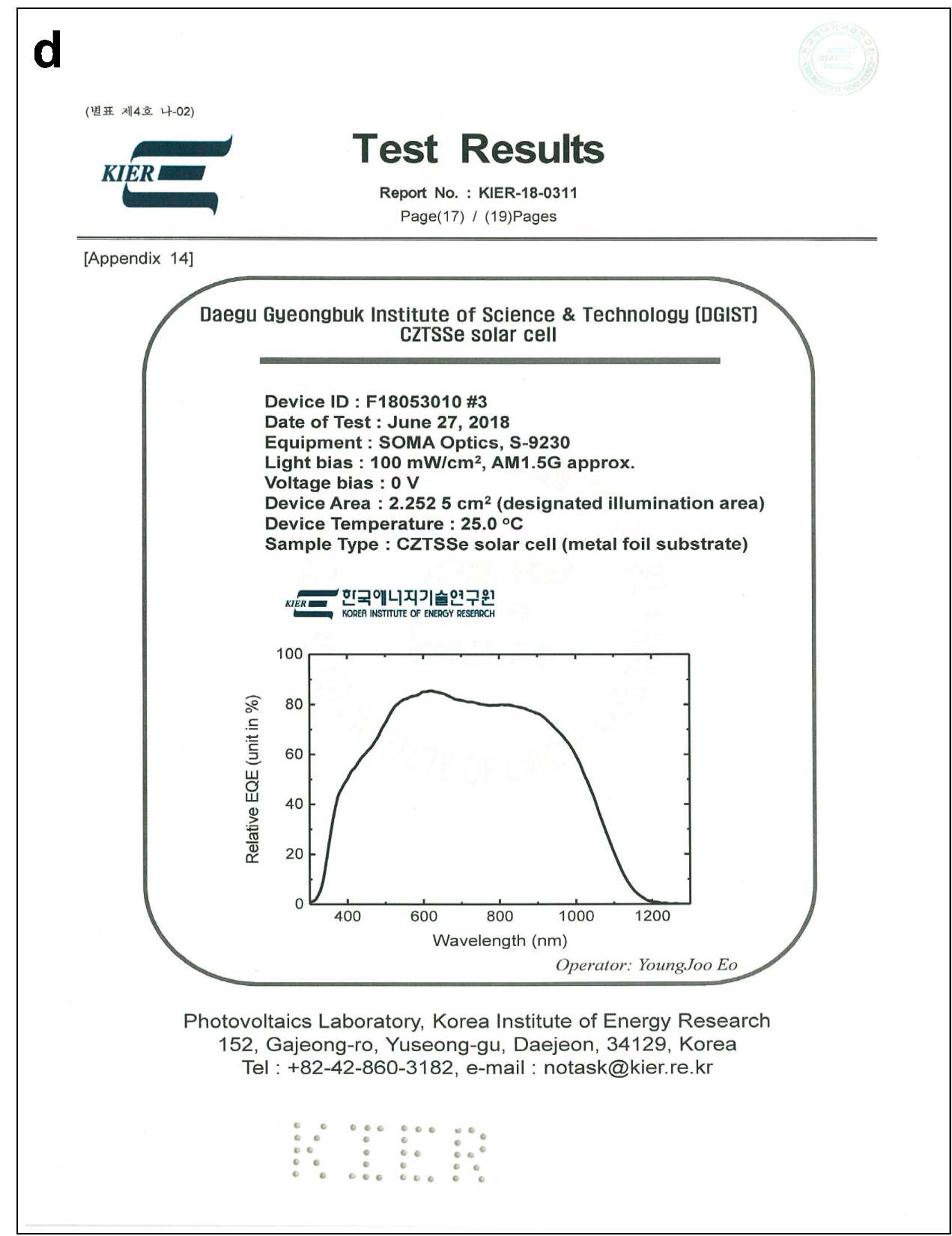


**Supplementary Figure. 7** *(continued)* **d** EQE characteristics of a 7.718% efficiency cell with an area of 2.252 cm^2^.


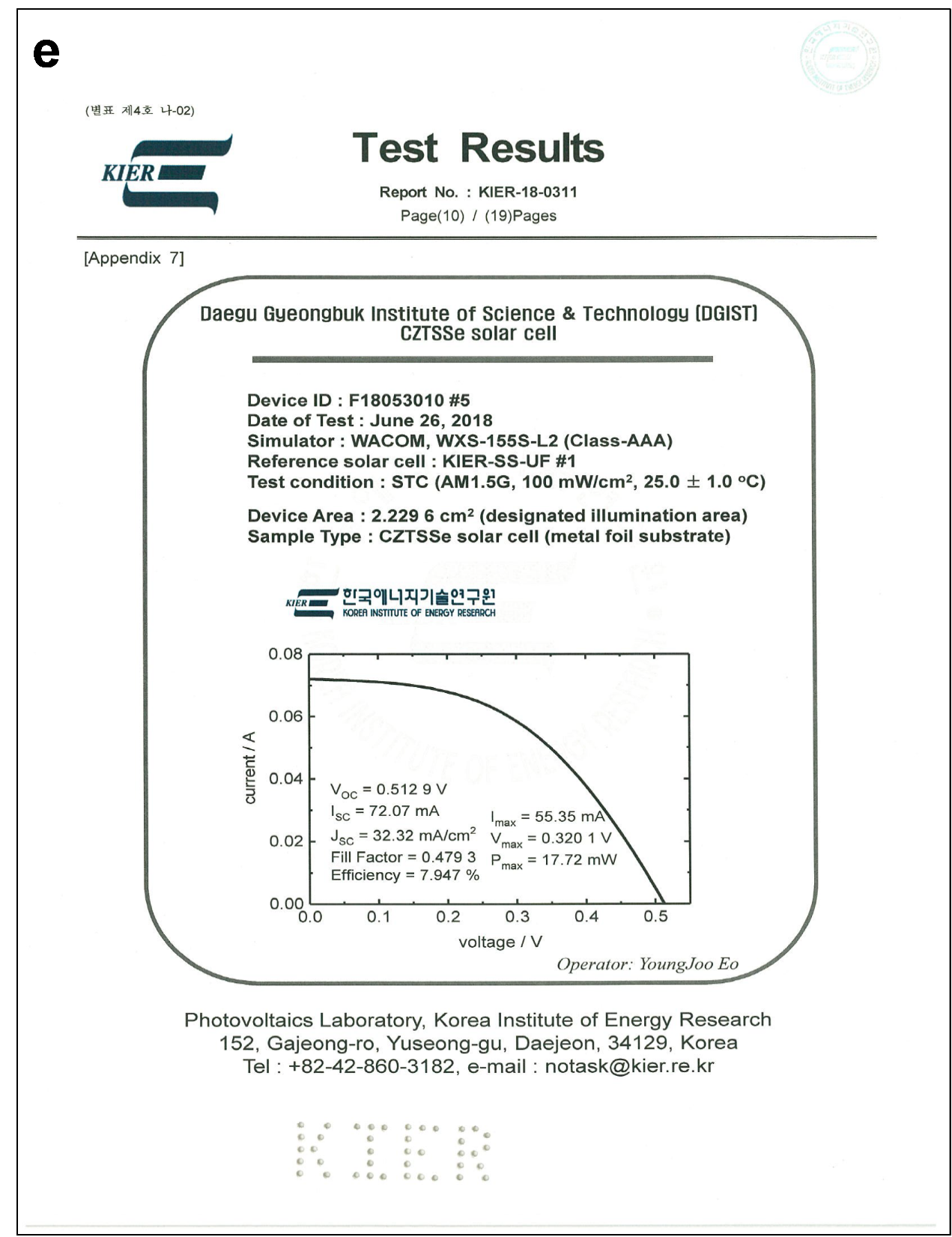


**Supplementary Figure. 7** *(continued)* **e** IV characteristics of a 7.947% efficiency cell with an area of 2.229 cm^2^.


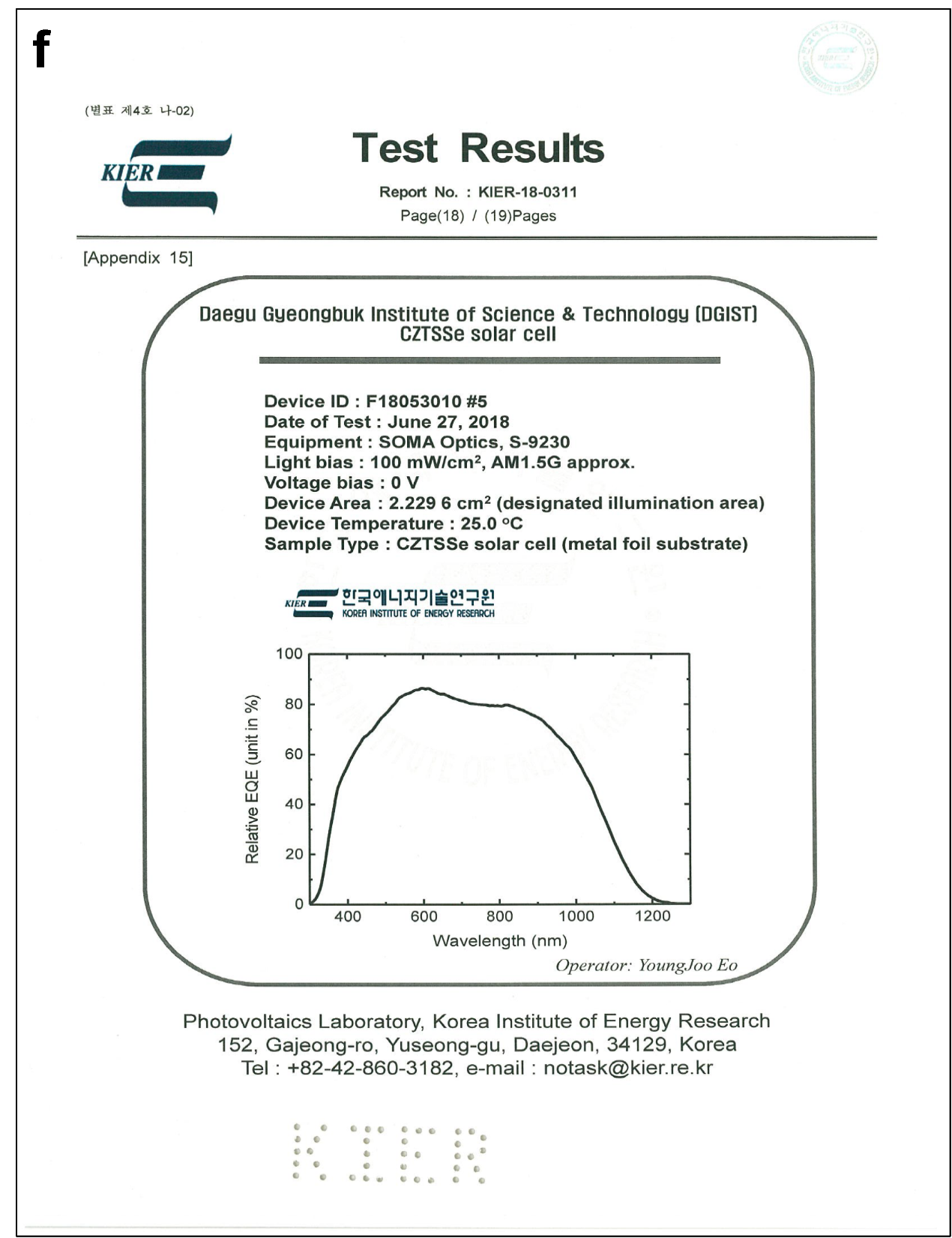


**Supplementary Figure. 7** *(continued)* **f** EQE characteristics of a 7.947% efficiency cell with an area of 2.229 cm^2^.

**
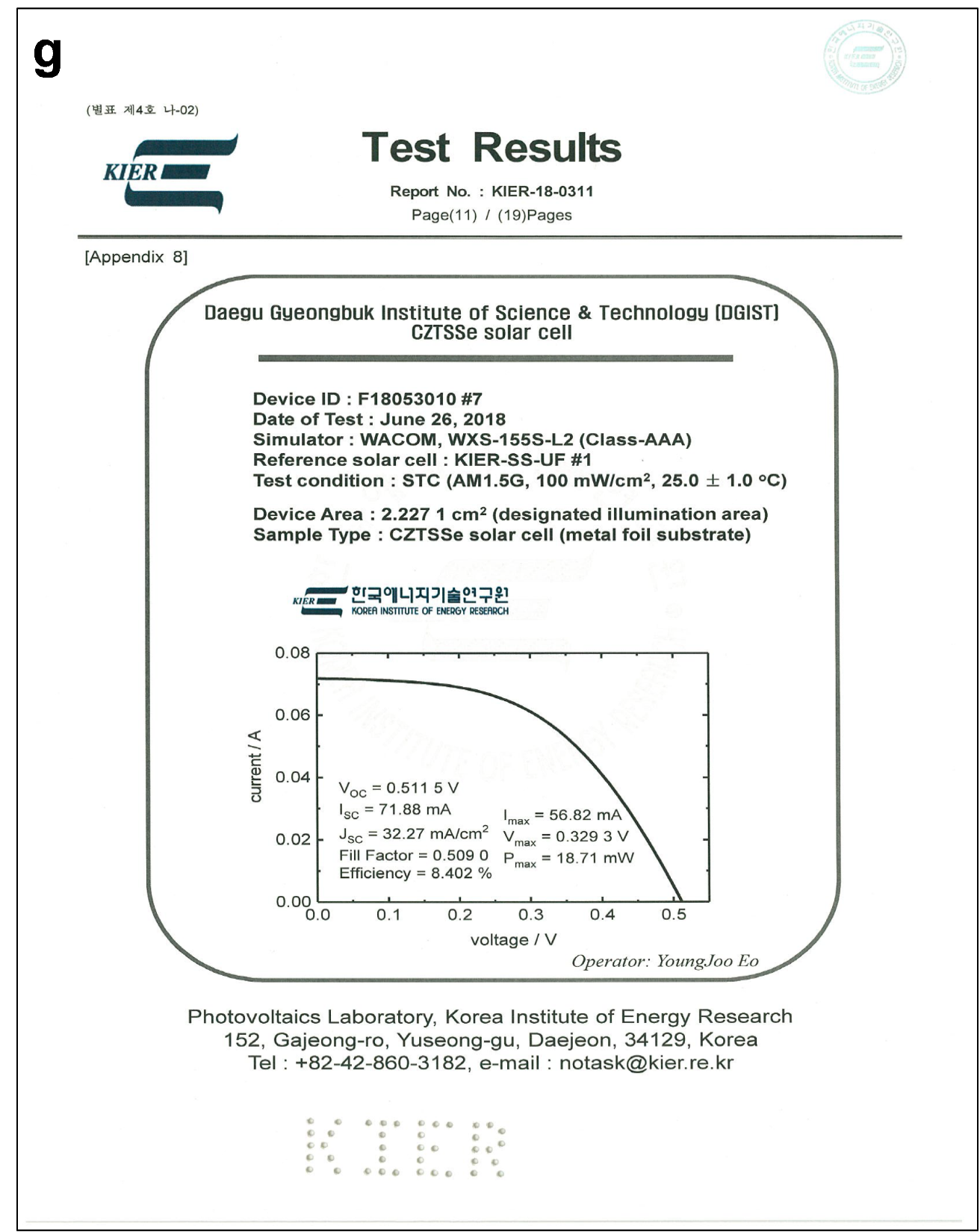
**

**Supplementary Figure. 7** *(continued)* **g** IV characteristics of an 8.402% efficiency cell with an area of 2.227 cm^2^.

**
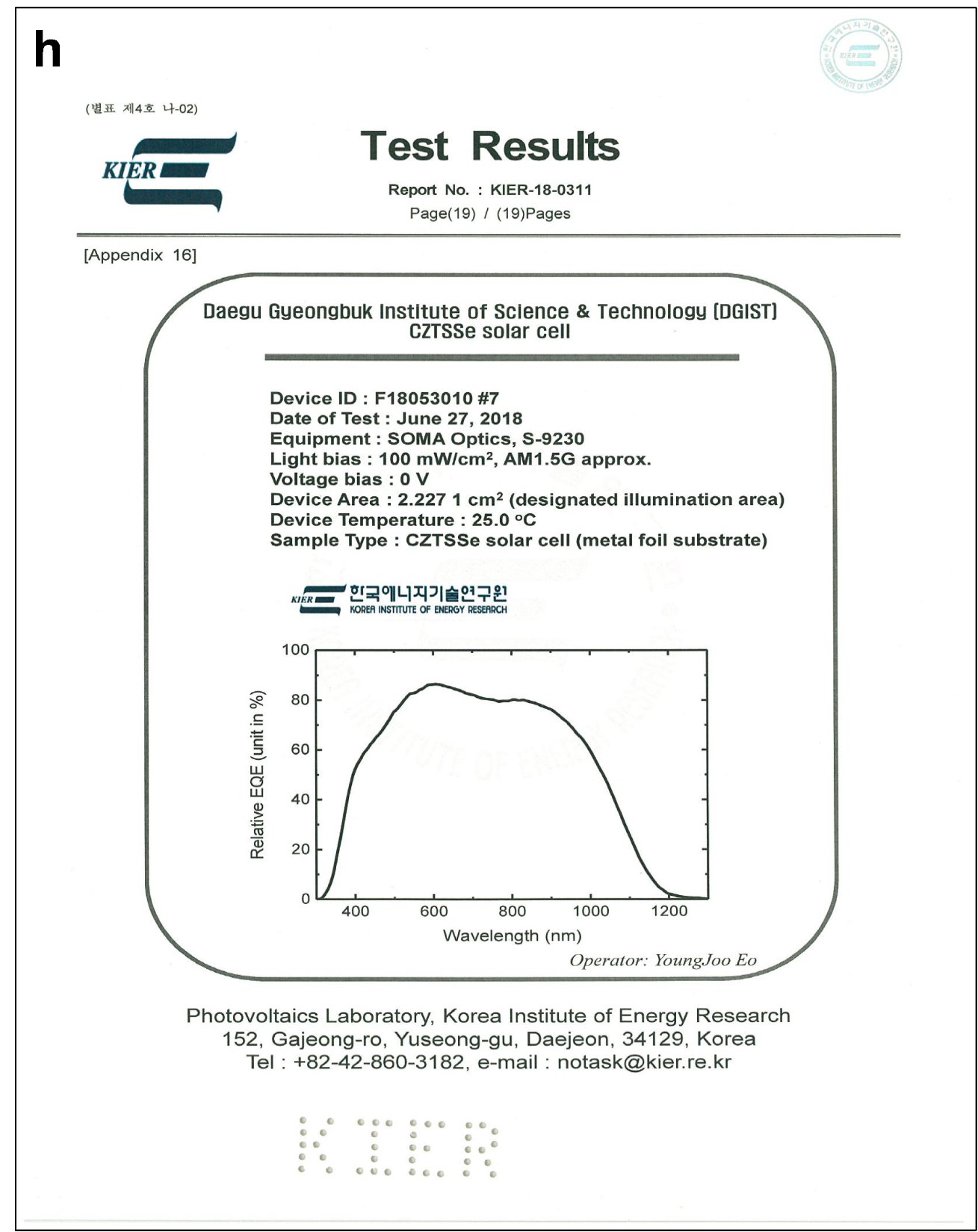
**

**Supplementary Figure. 7** *(continued)* **h** EQE characteristics of an 8.402% efficiency cell with an area of 2.227 cm^2^.

**
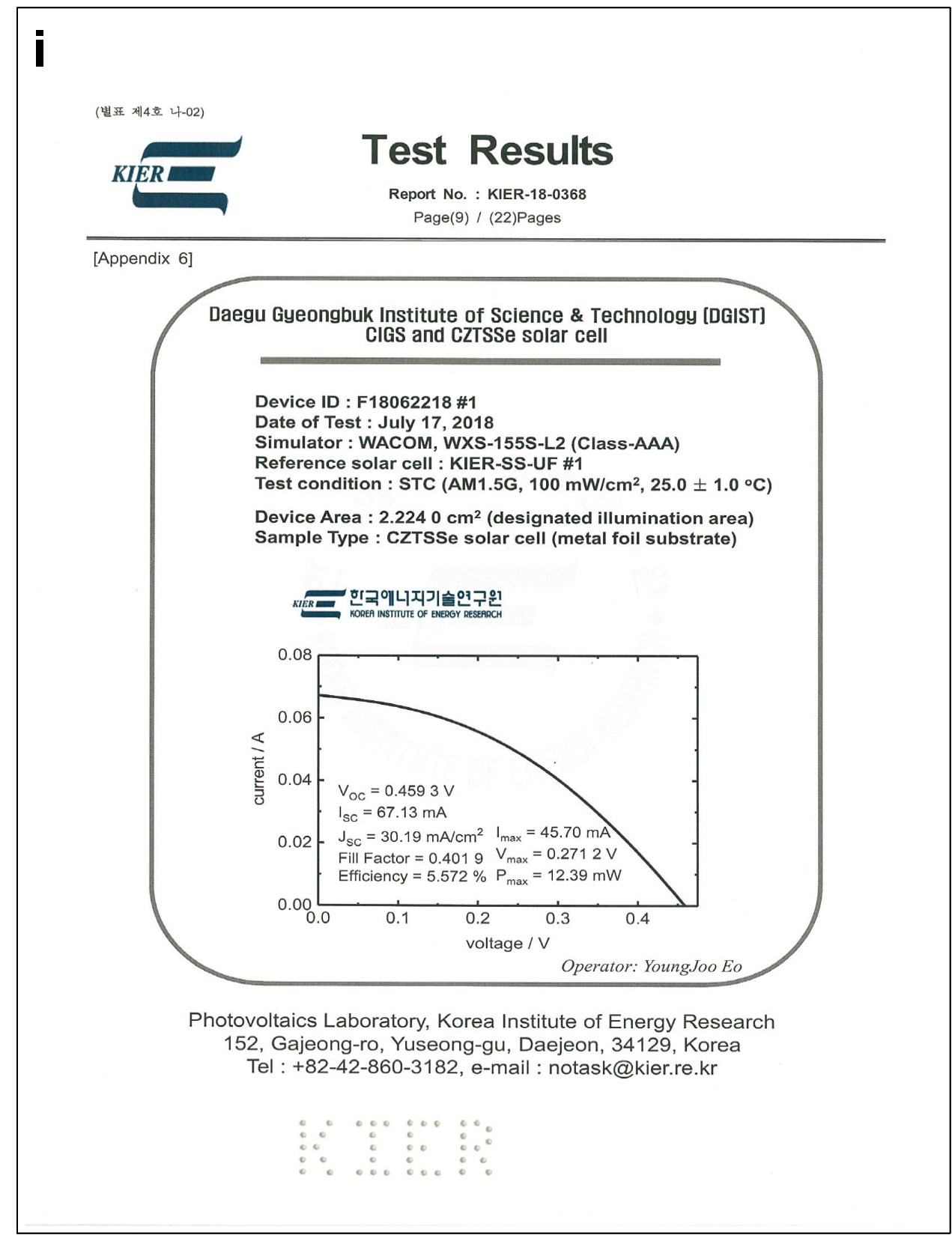
**

**Supplementary Figure. 7** *(continued)* **i** IV characteristics a 5.572% efficiency cell with an area of 2.224 cm^2^.

**
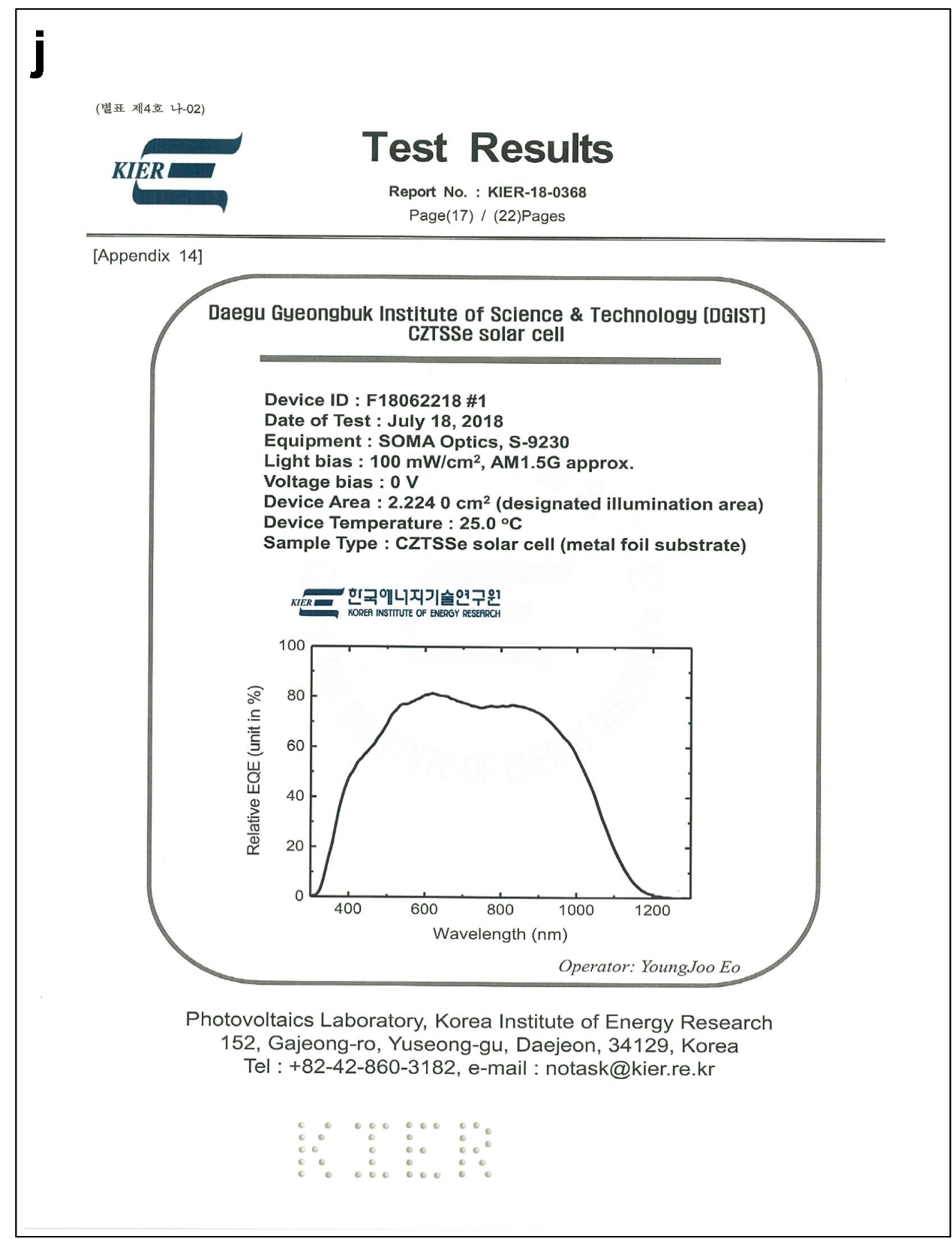
**

**Supplementary Figure. 7** *(continued)* **j** EQE characteristics of a 5.572% efficiency cell with an area of 2.224 cm^2^.


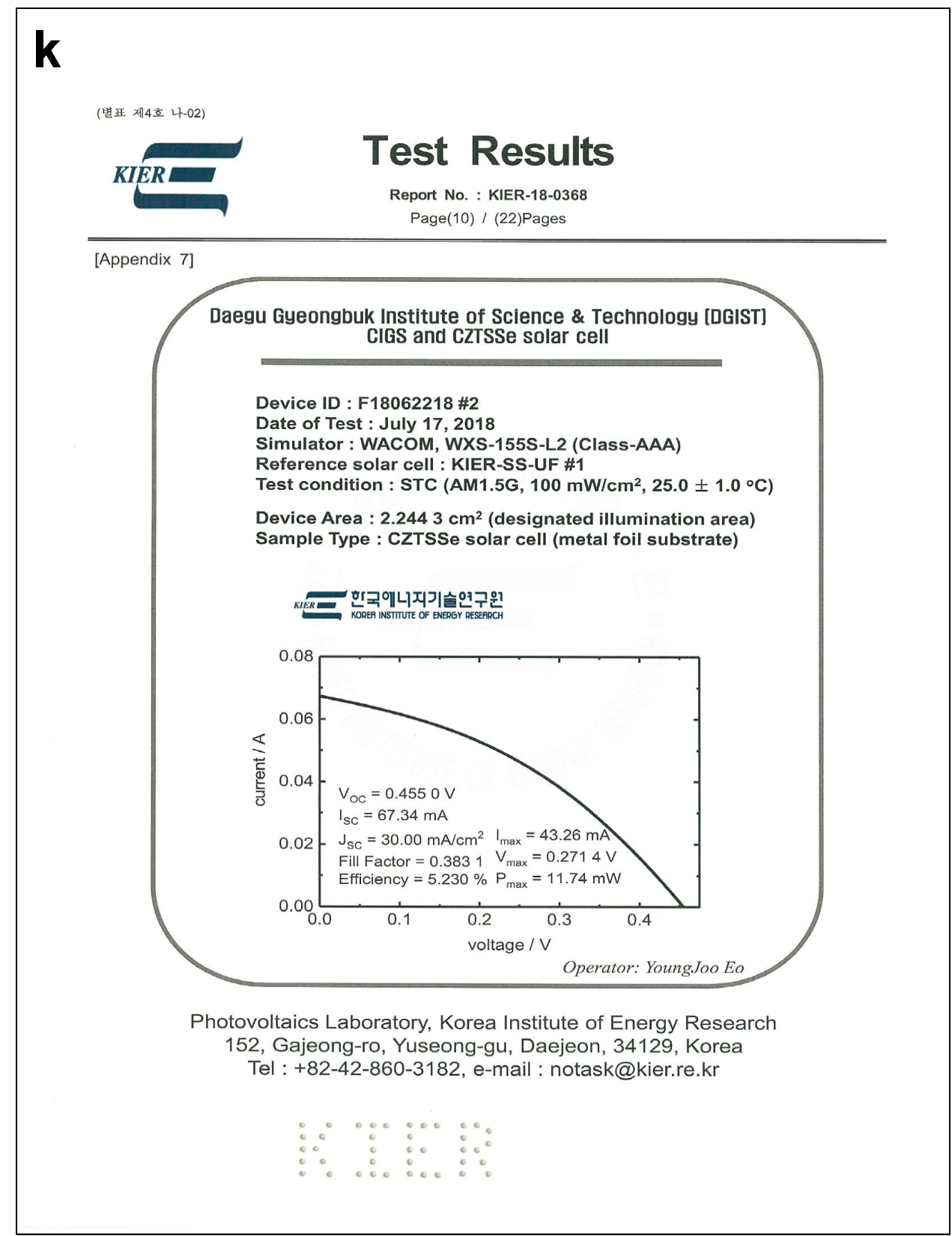


**Supplementary Figure. 7** *(continued)* **k** IV characteristics of a 5.230% efficiency cell with an area of 2.244 cm^2^.


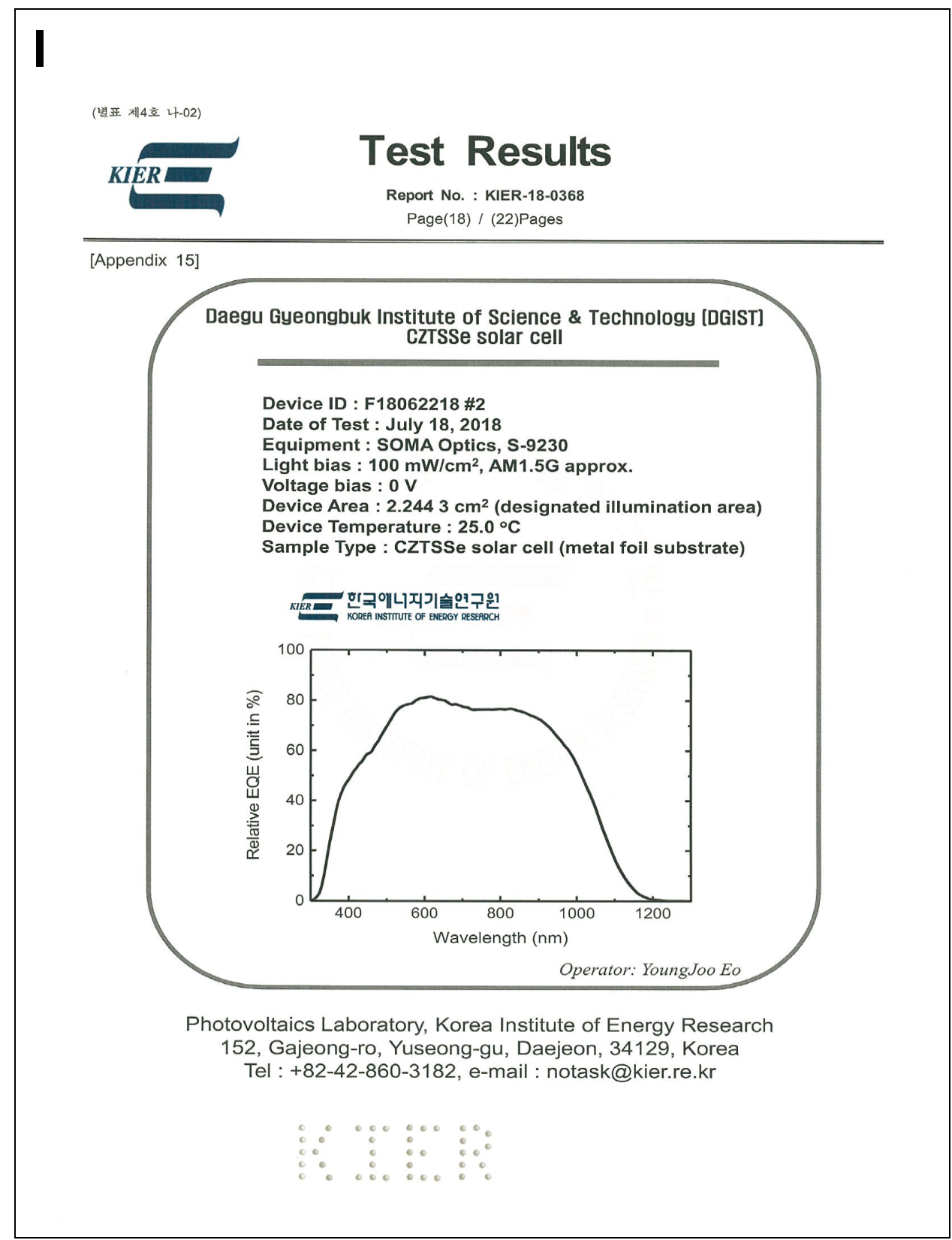


**Supplementary Figure. 7** *(continued)* **l** EQE characteristics of a 5.230% efficiency cell with an area of 2.244 cm^2^.


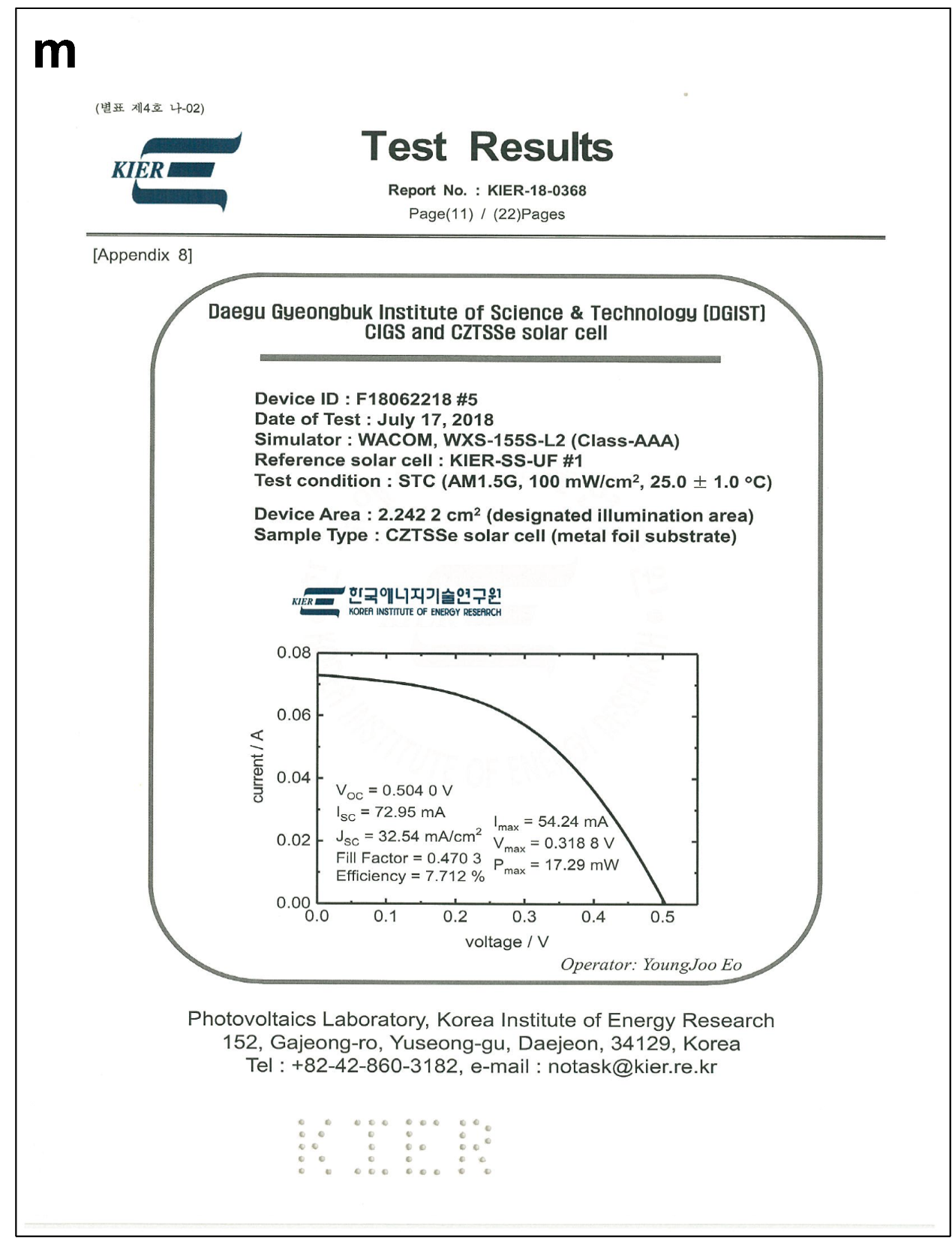


**Supplementary Figure. 7** *(continued)* **m** IV characteristics of a 7.712% efficiency cell with an area of 2.242 cm^2^.


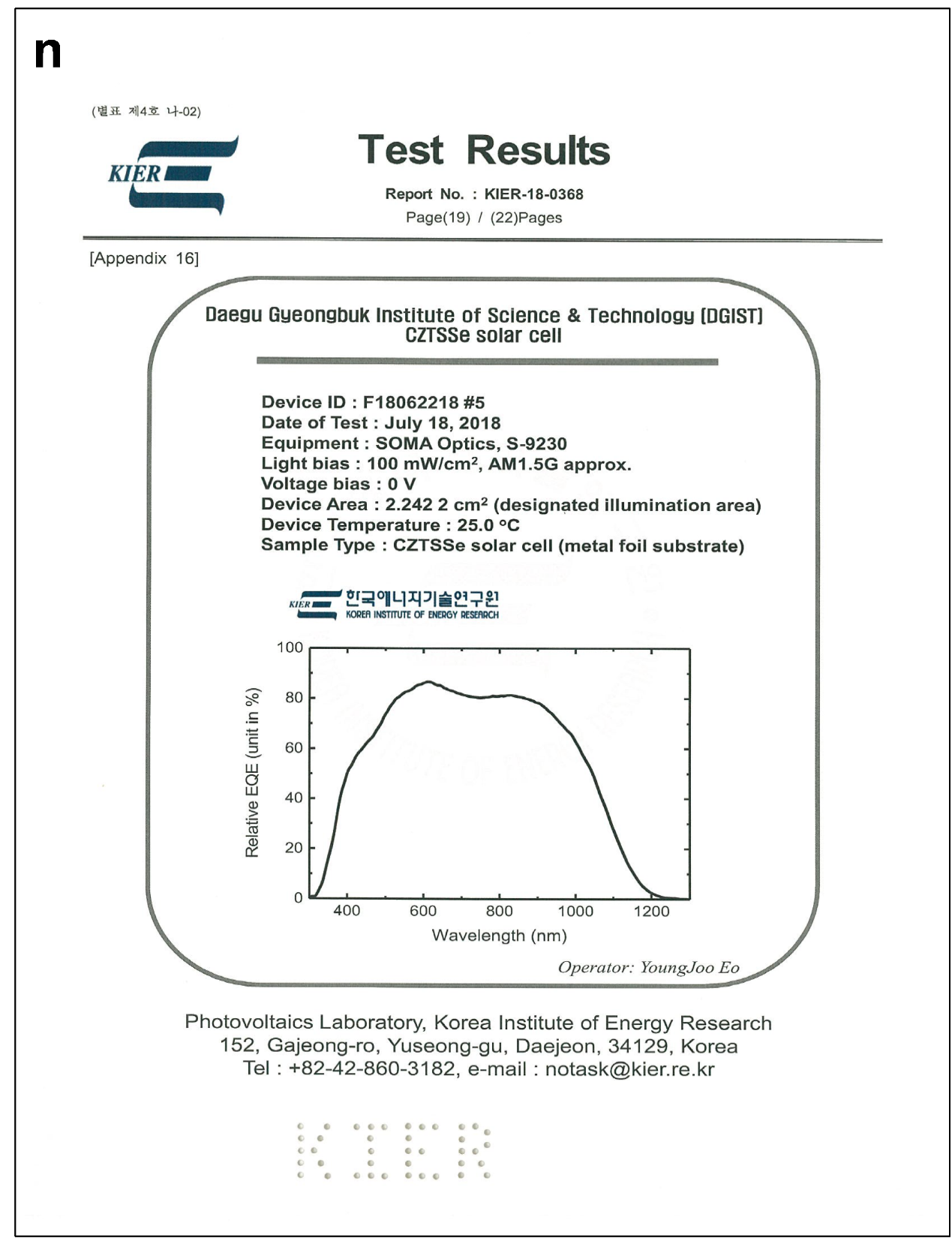


**Supplementary Figure. 7** *(continued)* **n** EQE characteristics of a 7.712% efficiency cell with an area of 2.242 cm^2^.


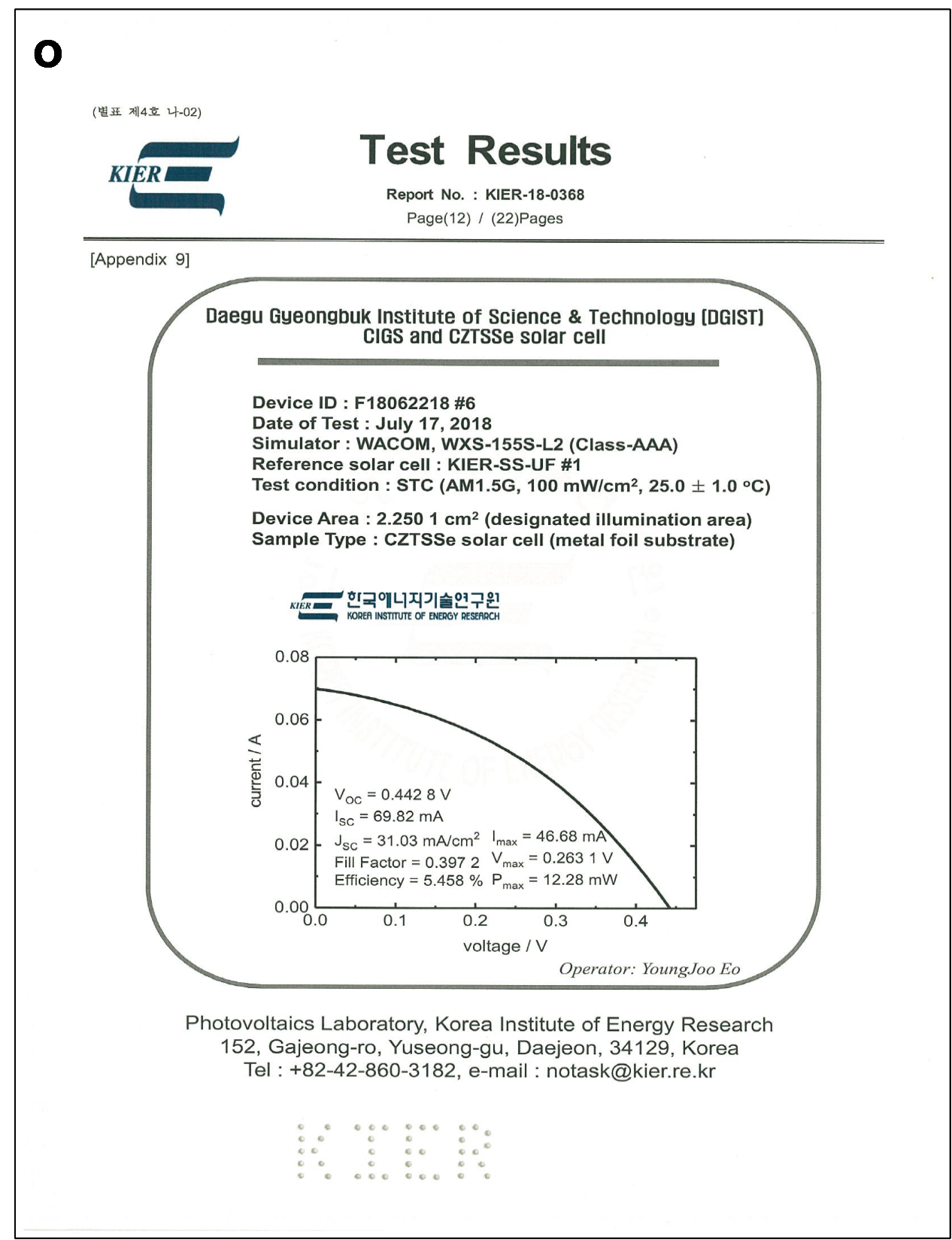


**Supplementary Figure. 7** *(continued)* **o** IV characteristics of a 5.458% efficiency cell with an area of 2.250 cm^2^.


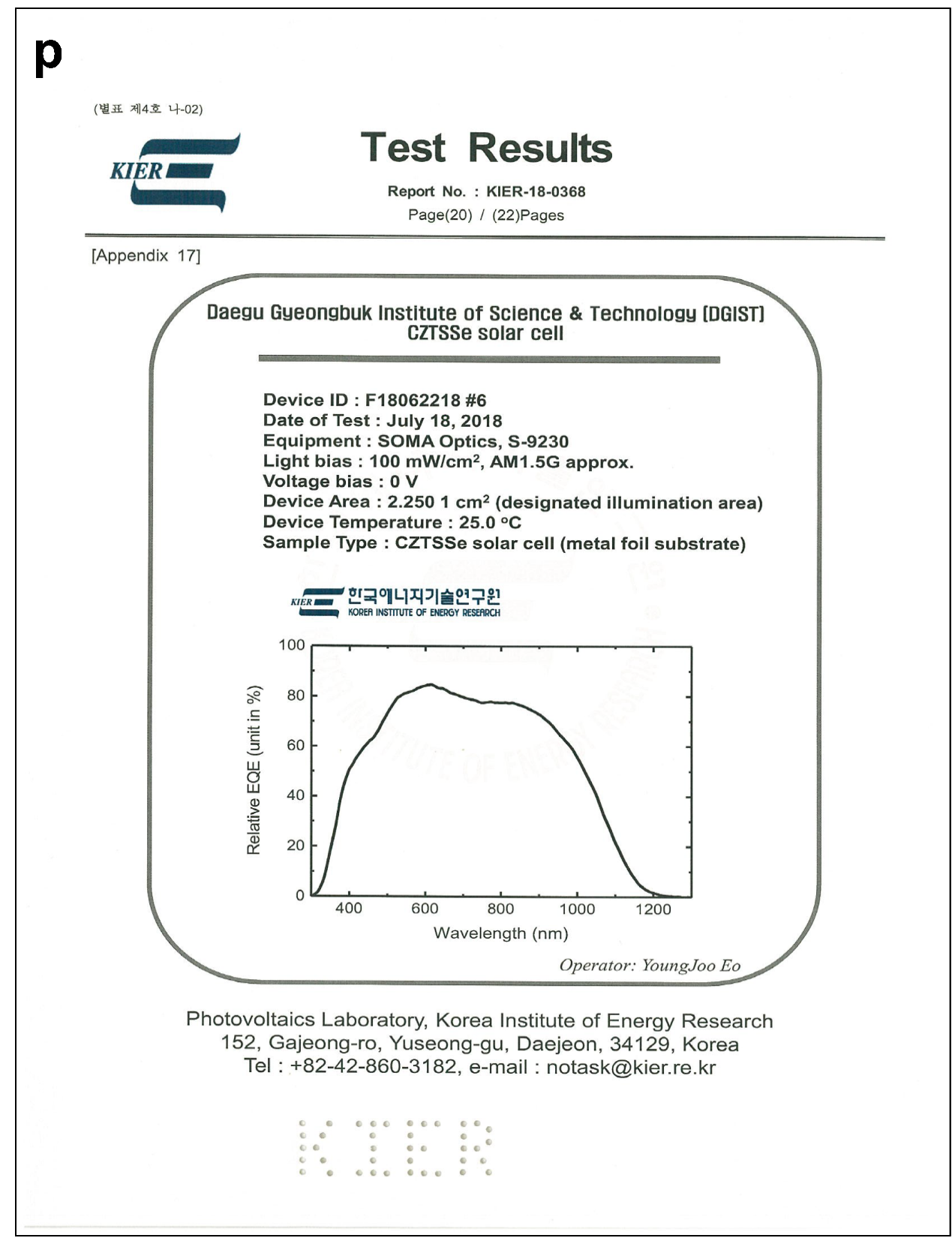


**Supplementary Figure. 7** *(continued)* **p** EQE characteristics of a 5.458% efficiency cell with an area of 2.250 cm^2^.


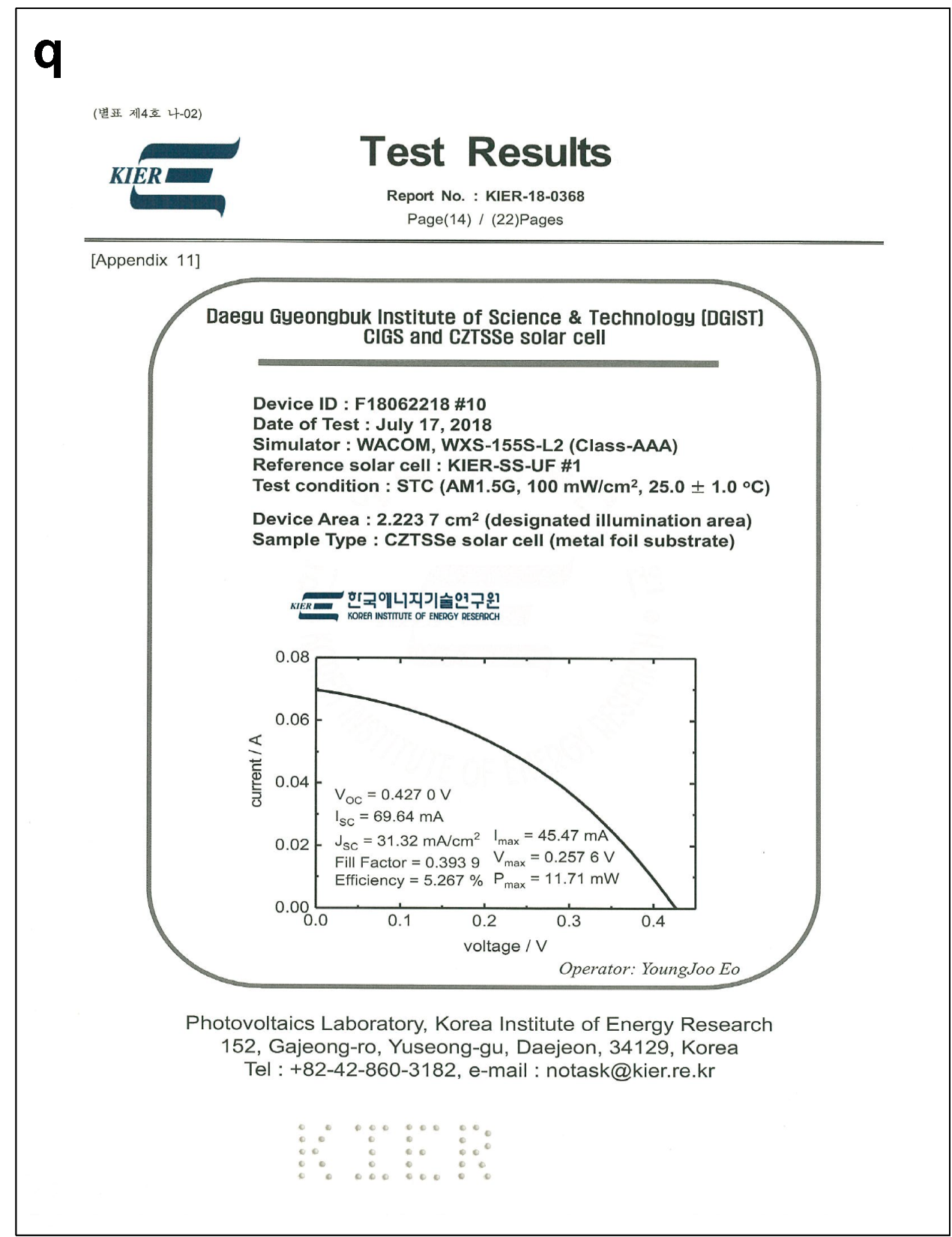


**Supplementary Figure. 7** *(continued)* **q** IV characteristics of a 5.267% efficiency cell with an area of 2.223 cm^2^.


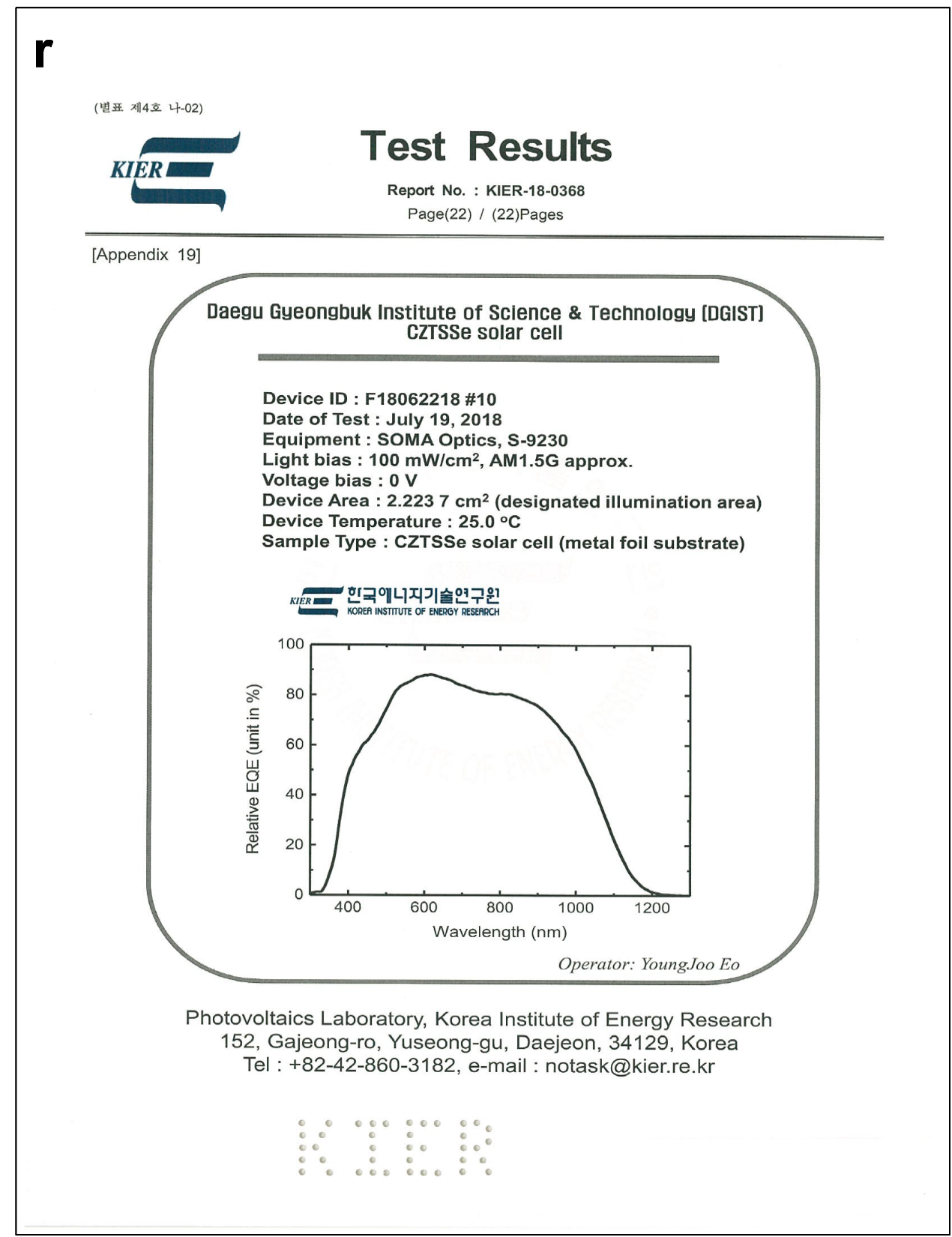


**Supplementary Figure. 7** *(continued)* **r** EQE characteristics of a 5.267% efficiency cell with an area of 2.223 cm^2^.


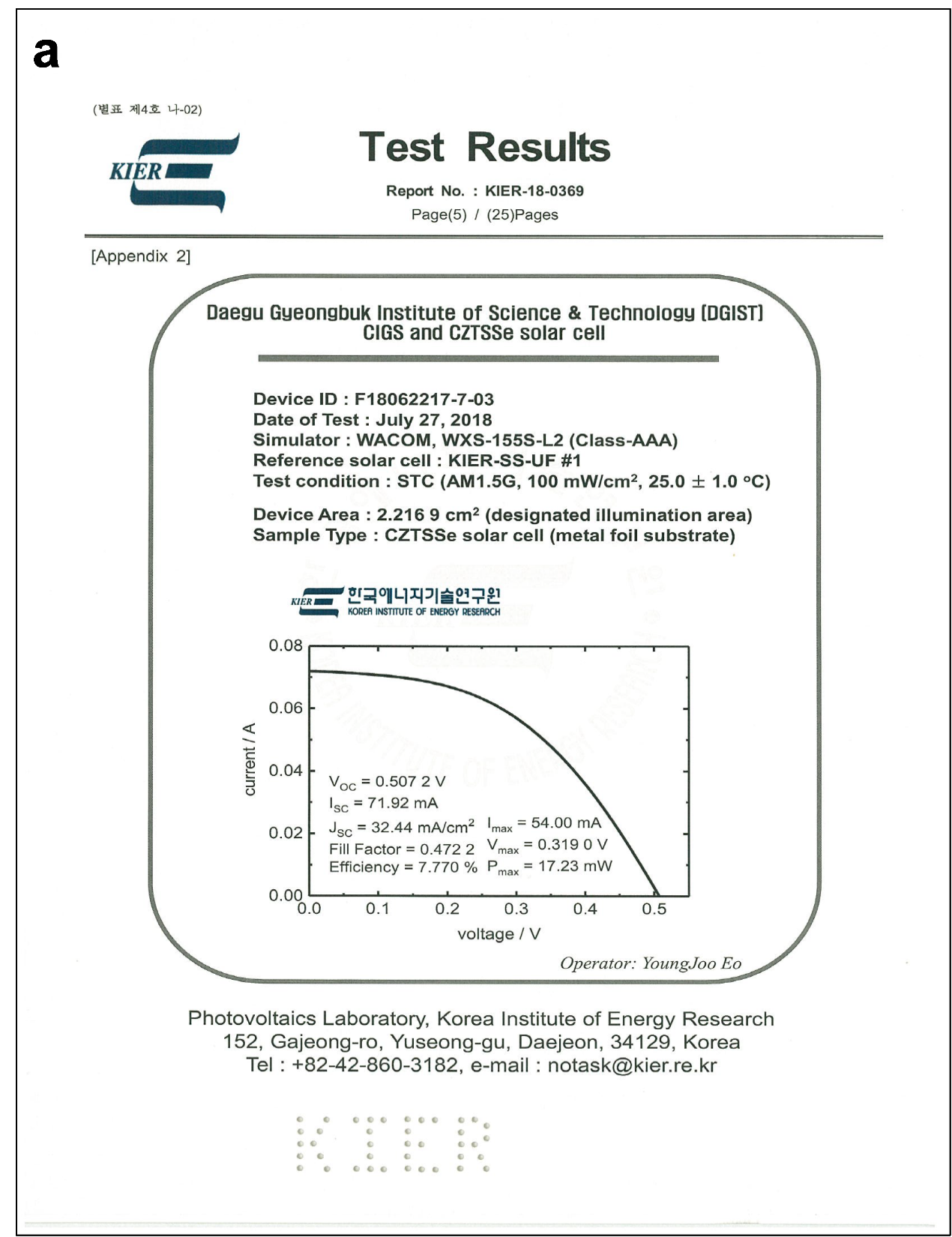


**Supplementary Figure. 8** The certificate of CZTSSe7-L flexible solar cells for 9 devices (from KIER). **a** IV characteristics of a 7.770% efficiency cell with an area of 2.216 cm^2^.


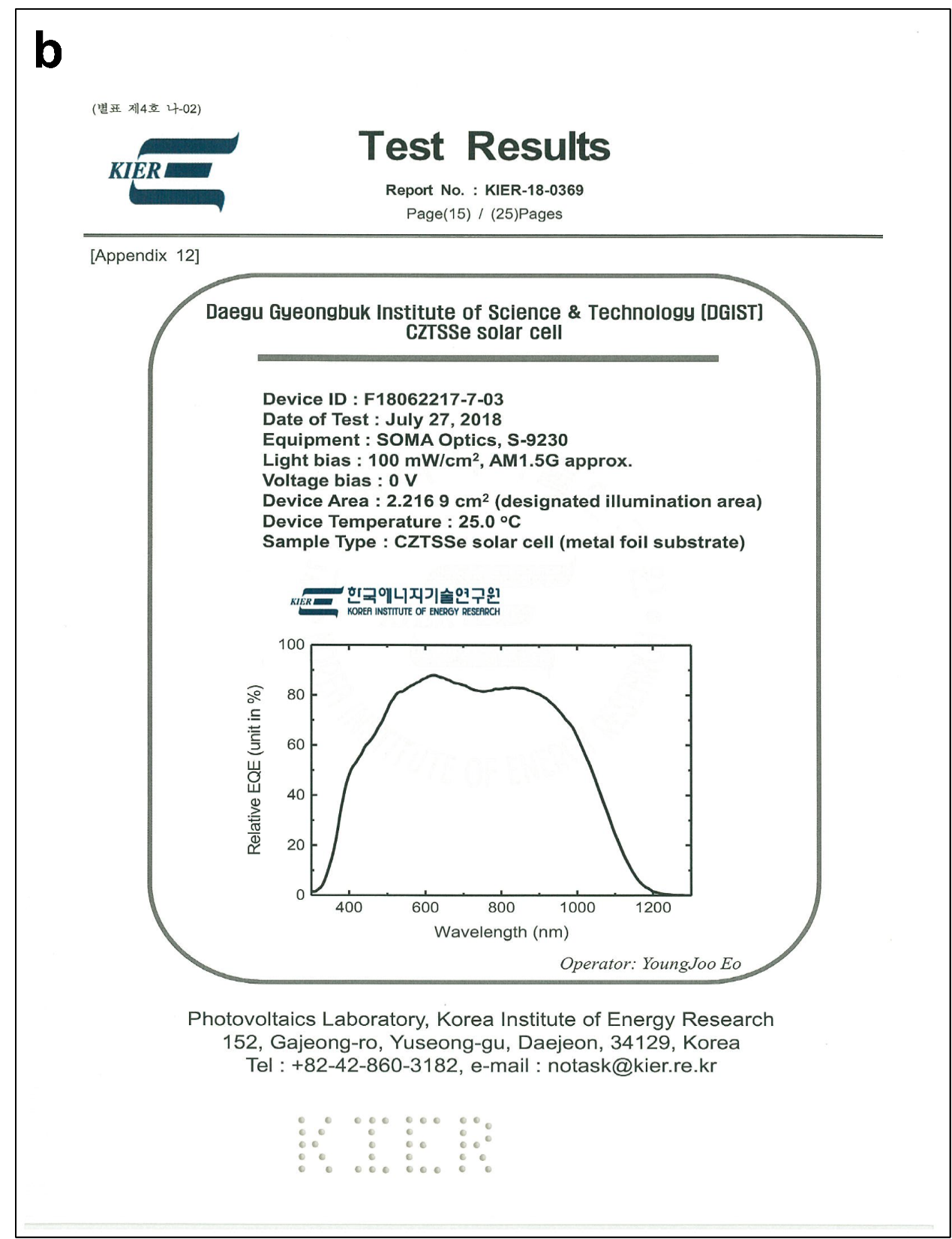


**Supplementary Figure. 8** *(continued)* **b** EQE characteristics of a 7.770% efficiency cell with an area of 2.216 cm^2^.


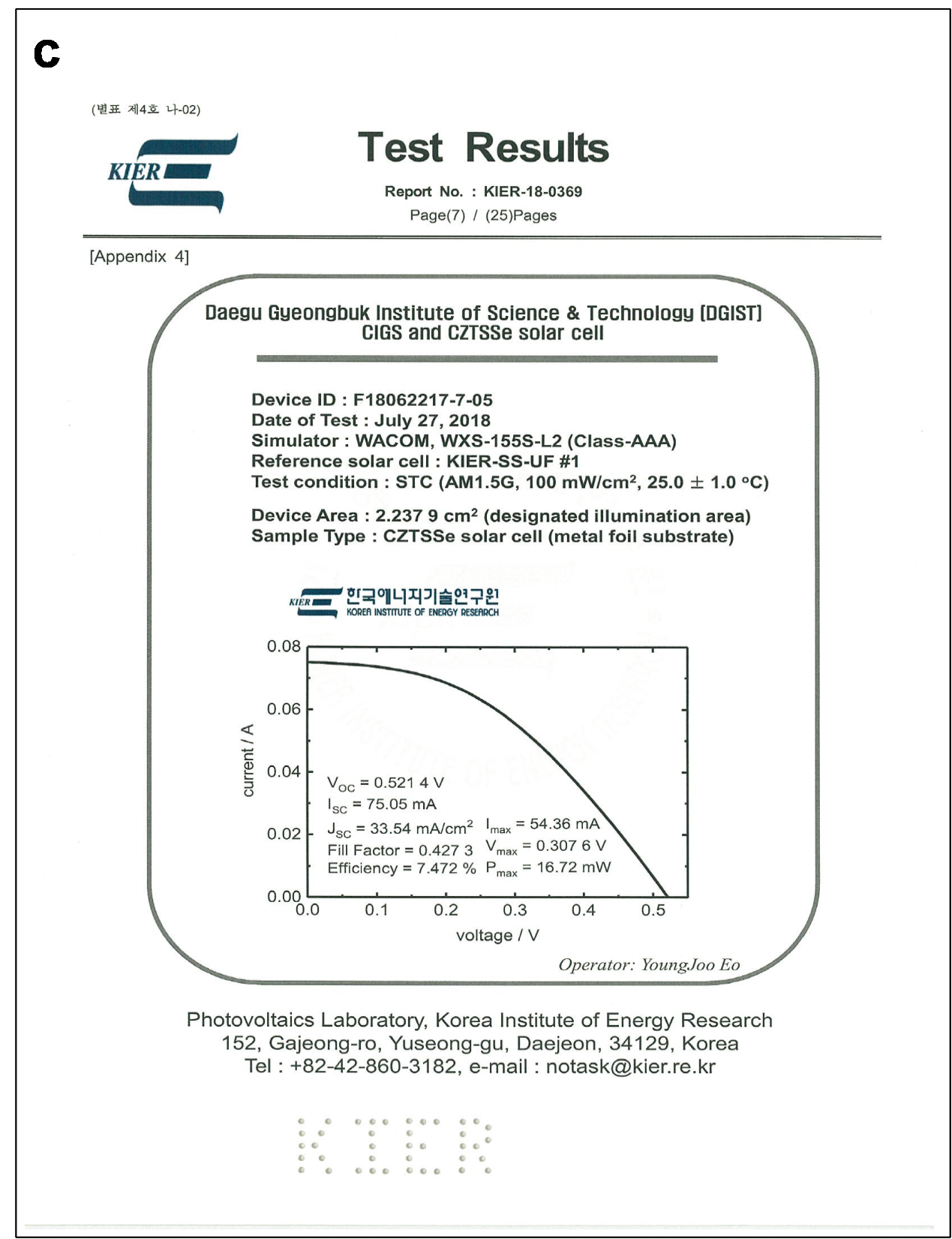


**Supplementary Figure. 8** *(continued)* **c** IV characteristics of a 7.472% efficiency cell with an area of 2.237 cm^2^.


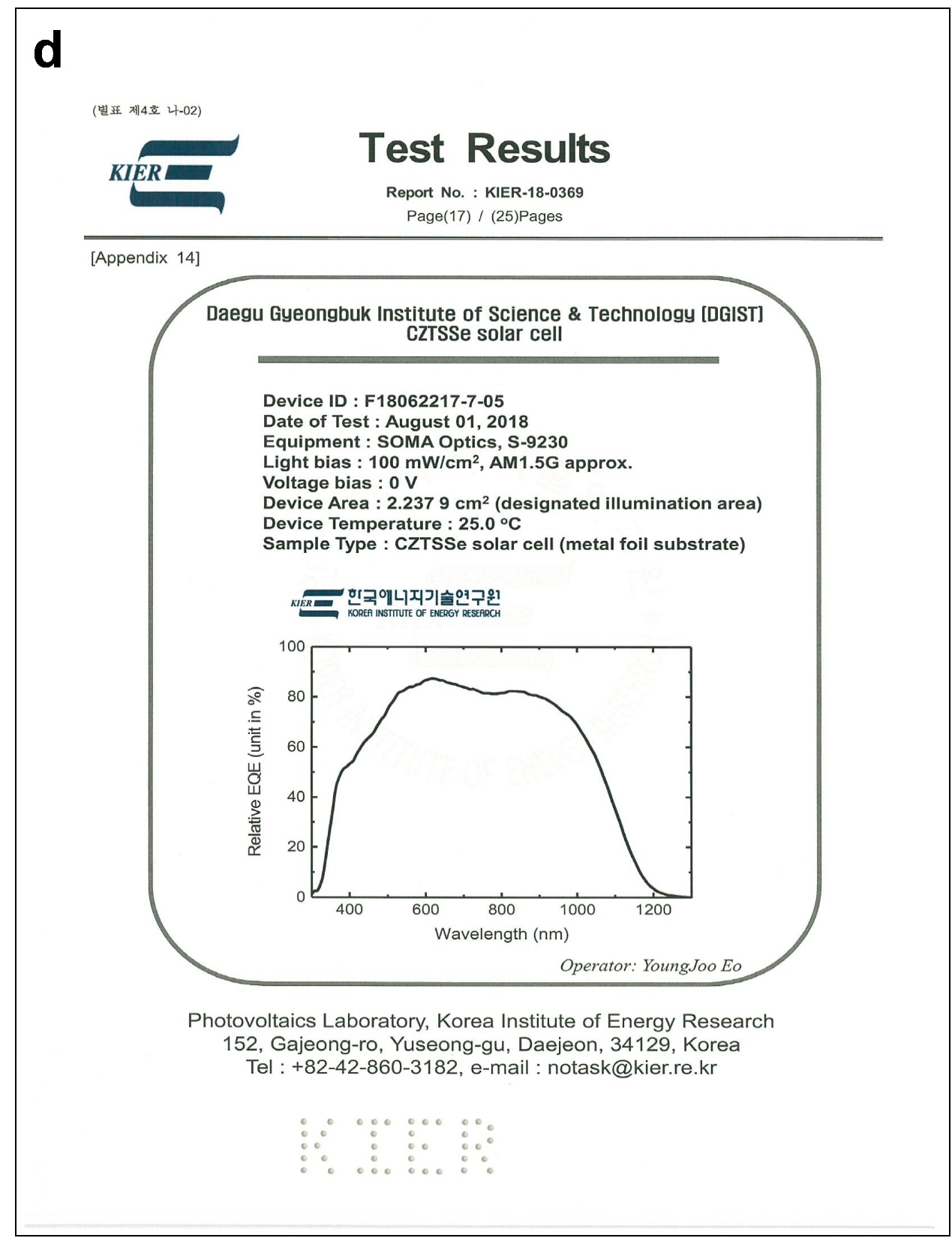


**Supplementary Figure. 8** *(continued)* **d** EQE characteristics of a 7.472% efficiency cell with an area of 2.237 cm^2^.


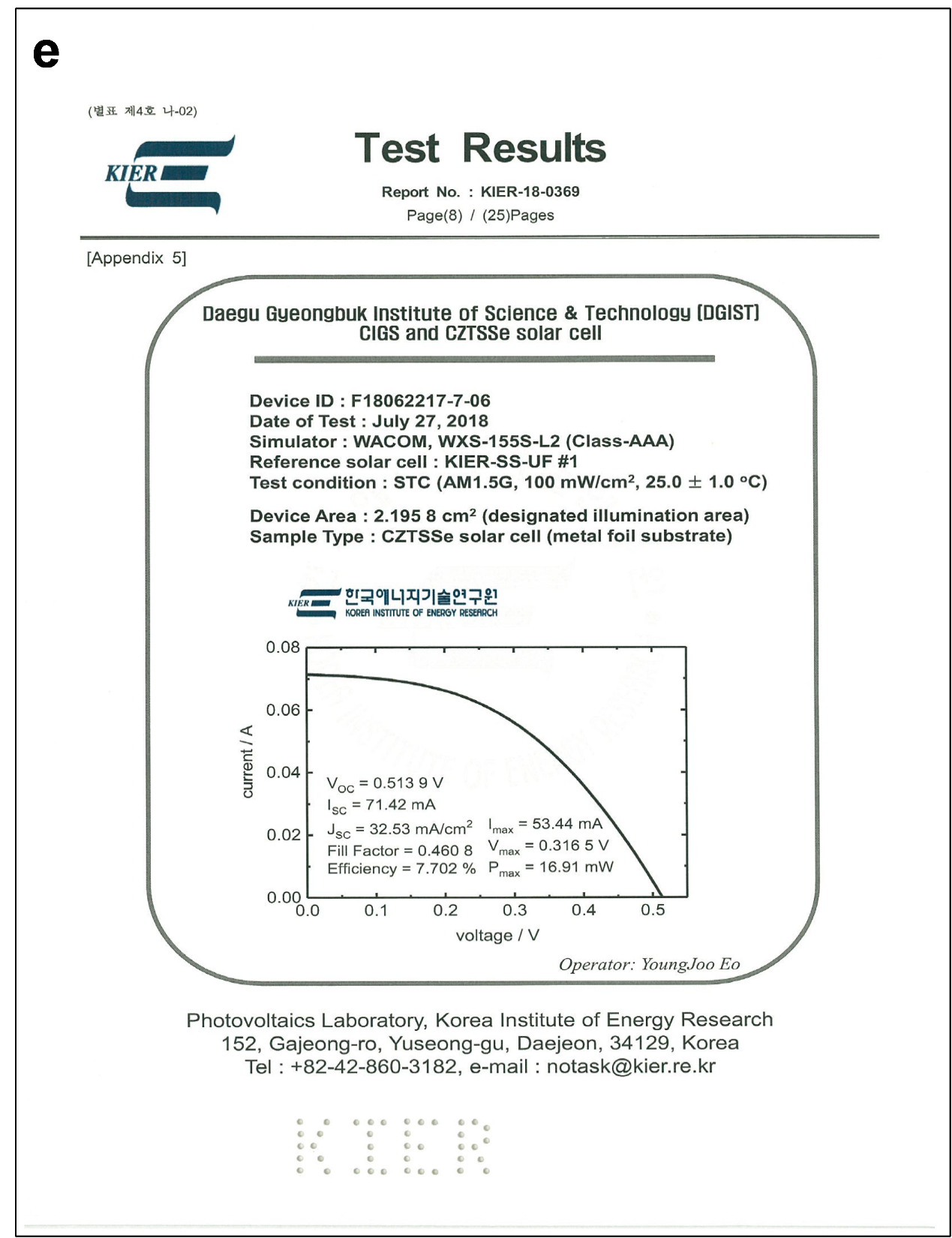


**Supplementary Figure. 8** *(continued)* **e** IV characteristics of a 7.702% efficiency cell with an area of 2.195 cm^2^.


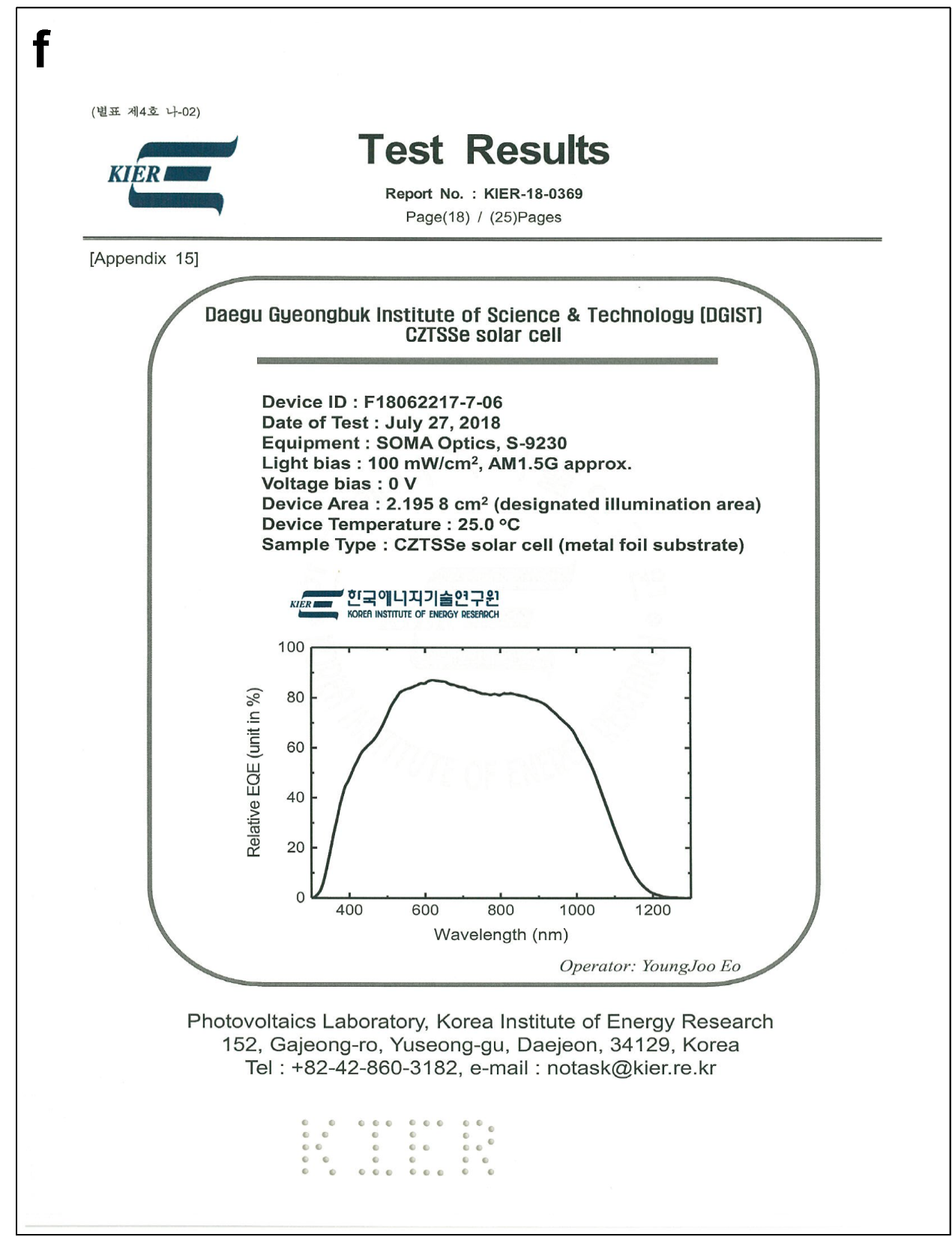


**Supplementary Figure. 8** *(continued)* **f** EQE characteristics of a 7.702% efficiency cell with an area of 2.195 cm^2^.


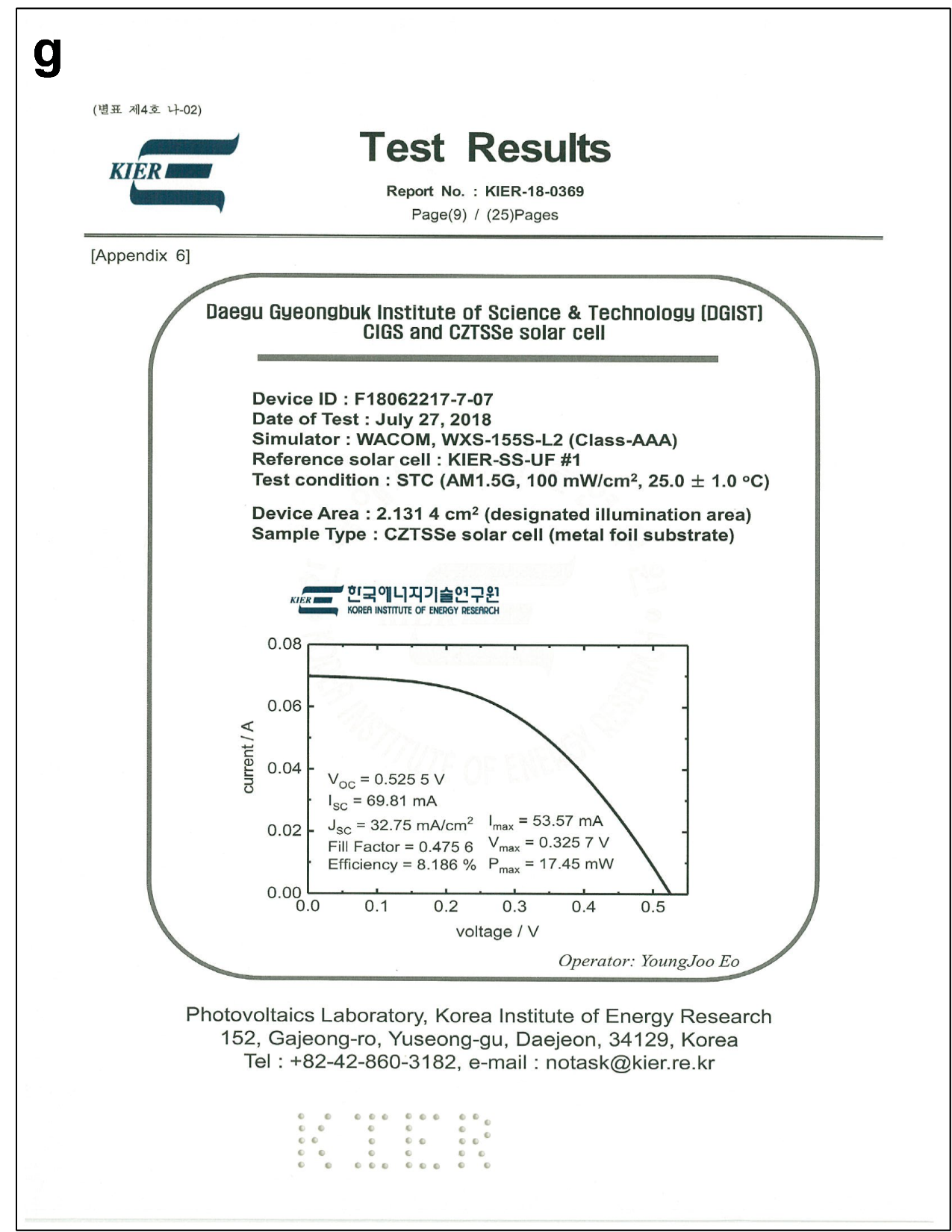


**Supplementary Figure. 8** *(continued)* **g** IV characteristics of an 8.186% efficiency cell with an area of 2.131 cm^2^.


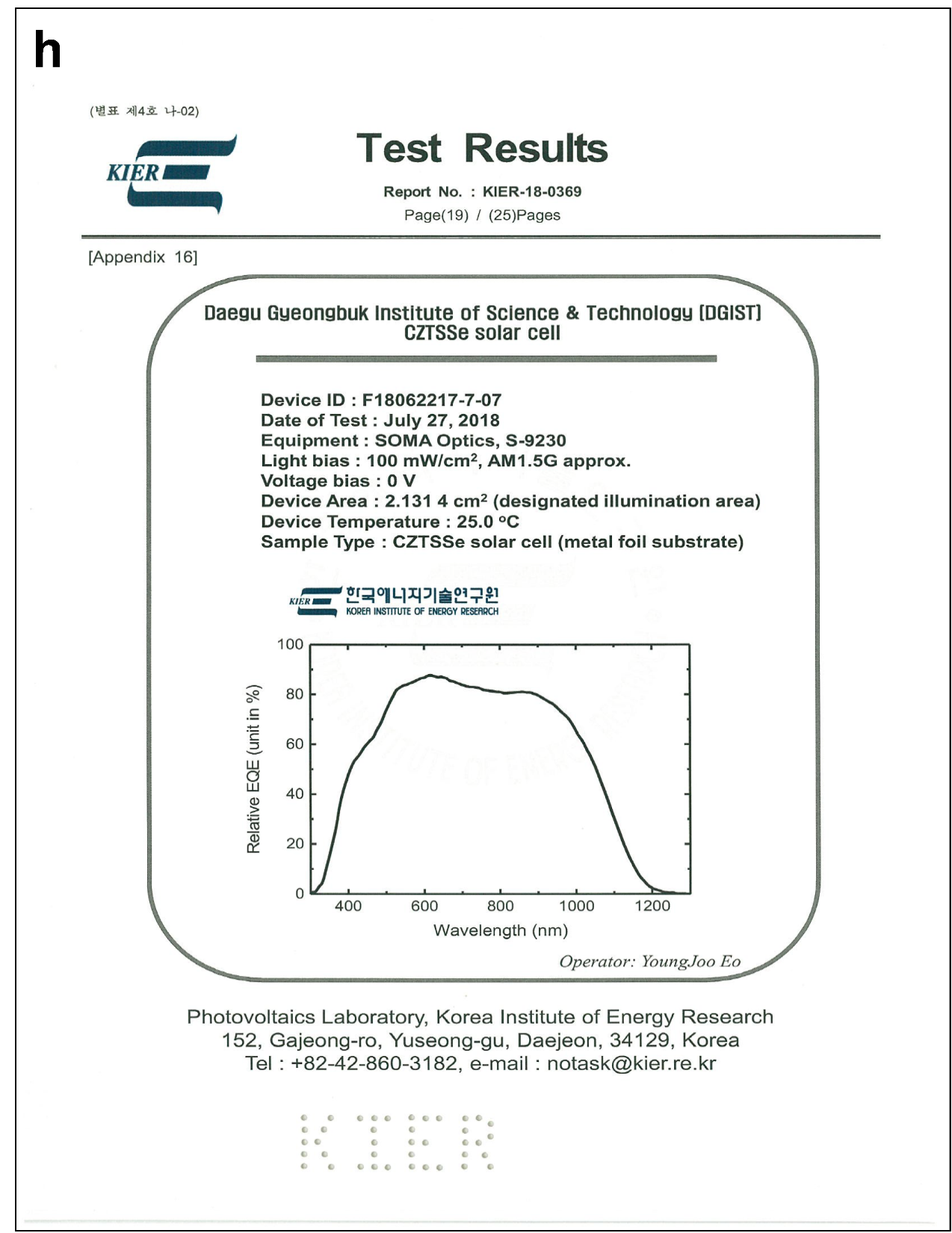


**Supplementary Figure. 8** *(continued)* **h** EQE characteristics of an 8.186% efficiency cell with an area of 2.131 cm^2^.


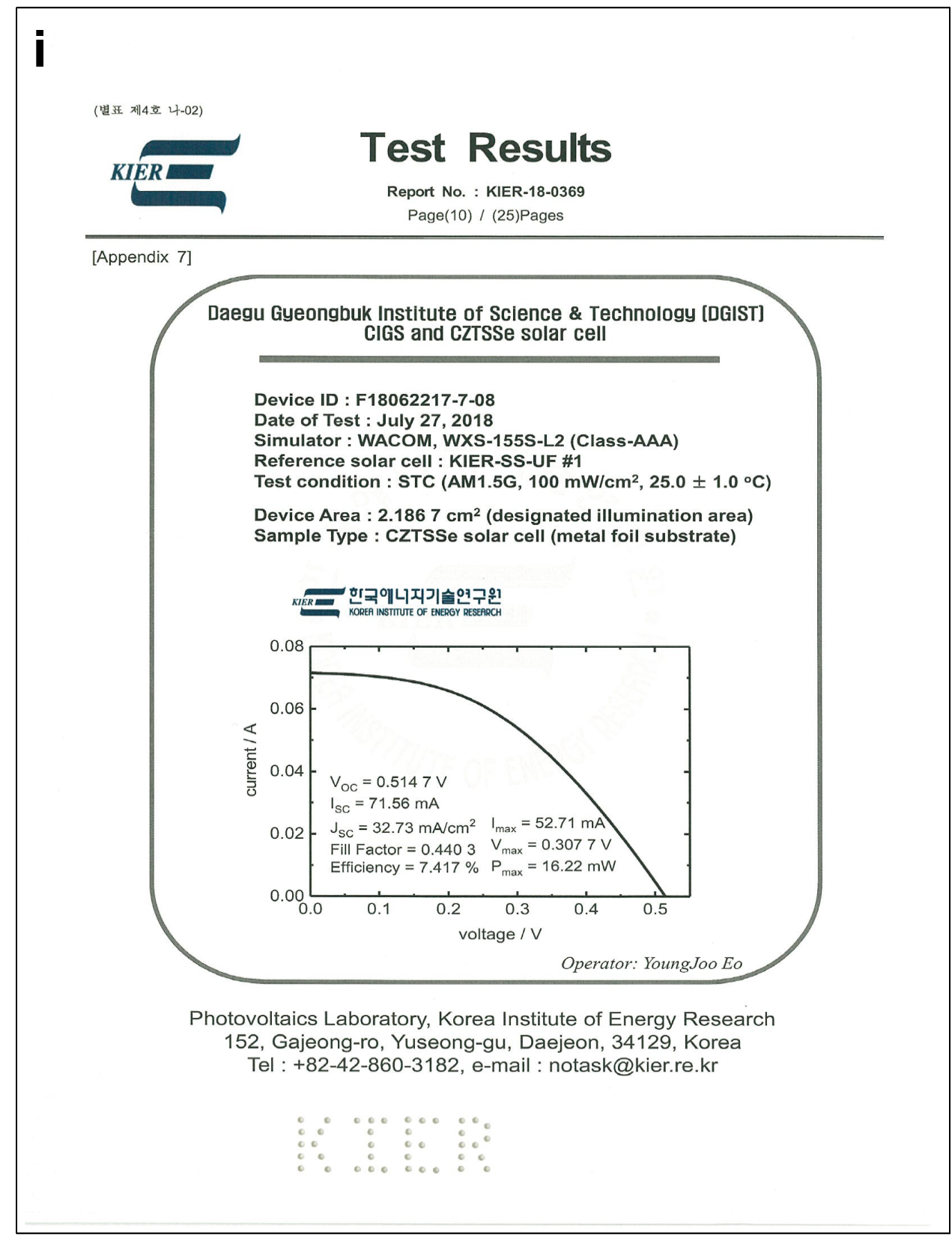


**Supplementary Figure. 8** *(continued)* **i** IV characteristics of a 7.417% efficiency cell with an area of 2.186 cm^2^.


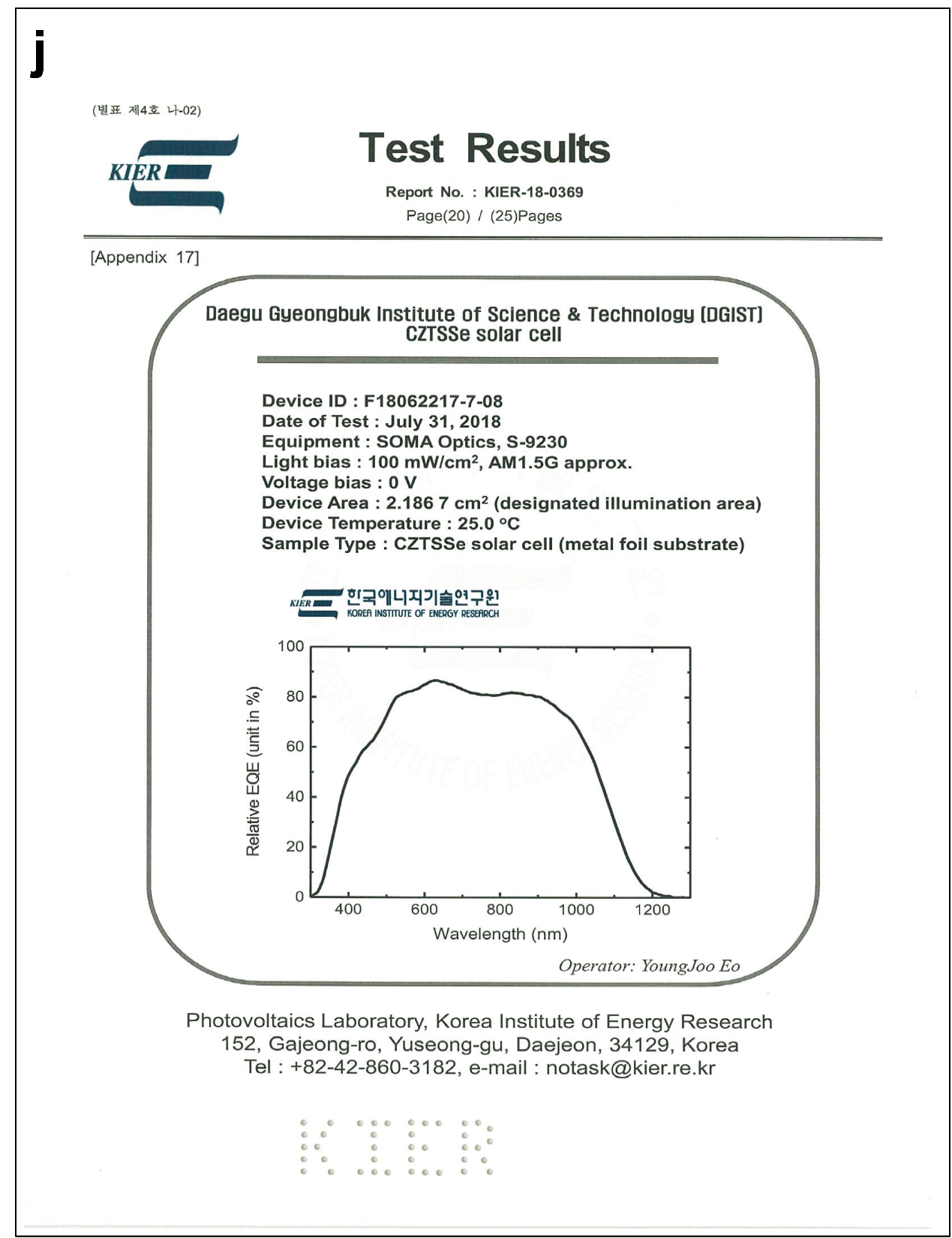


**Supplementary Figure. 8** *(continued)* **j** EQE characteristics of a 7.417% efficiency cell with an area of 2.186 cm^2^.


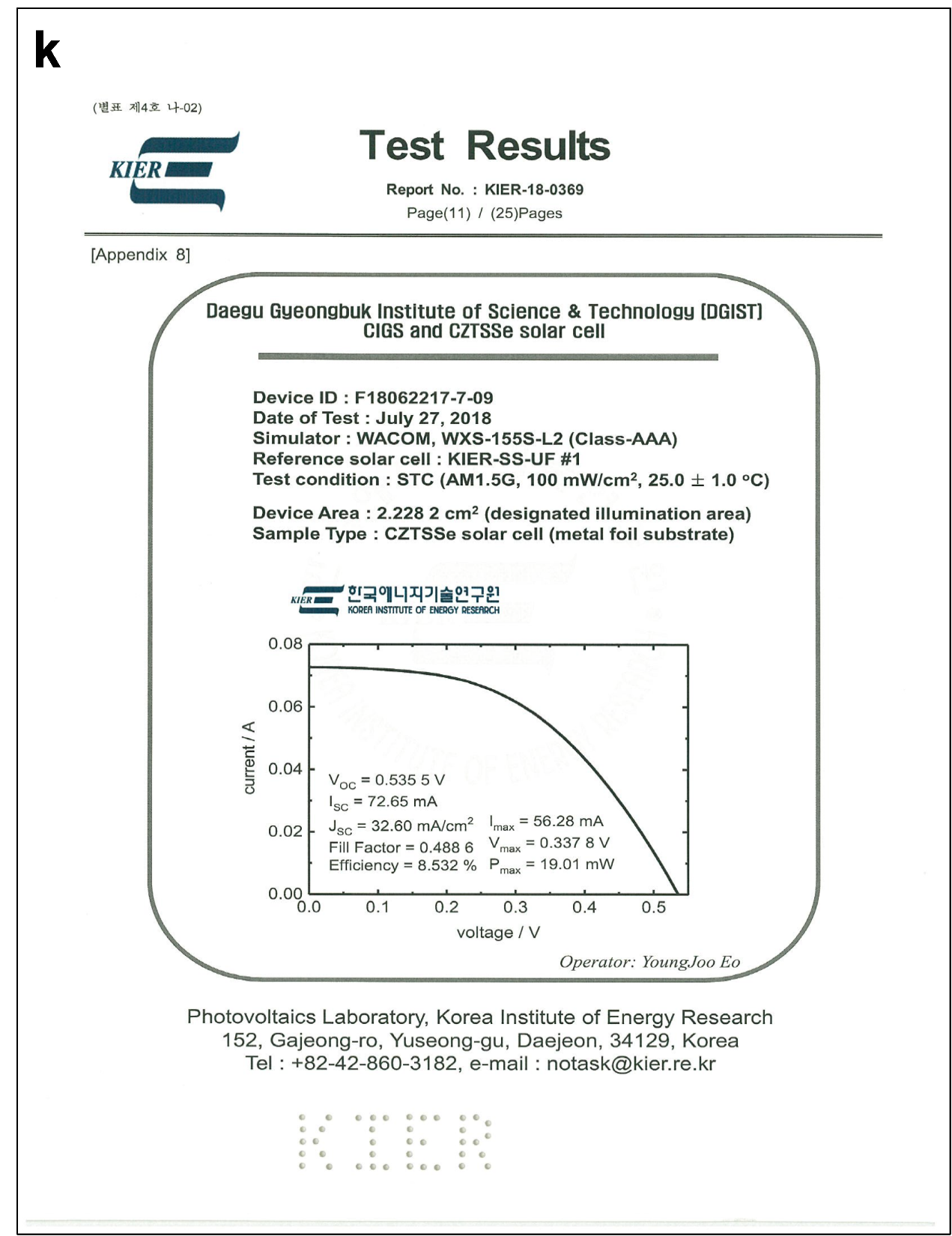


**Supplementary Figure. 8** *(continued)* **k** IV characteristics of an 8.532% efficiency cell with an area of 2.228 cm^2^.


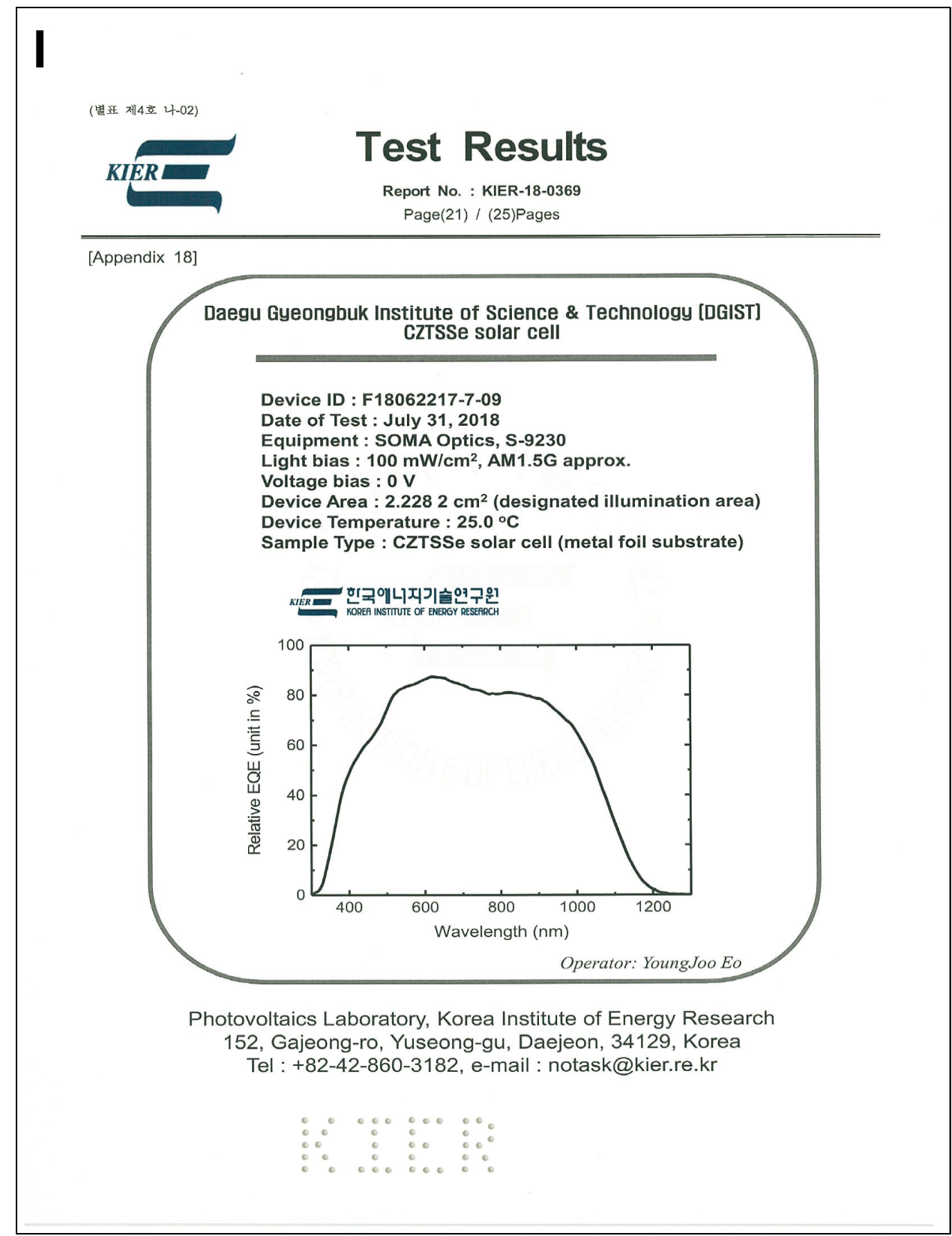


**Supplementary Figure. 8** *(continued)* **l** EQE characteristics of an 8.532% efficiency cell with an area of 2.228 cm^2^.


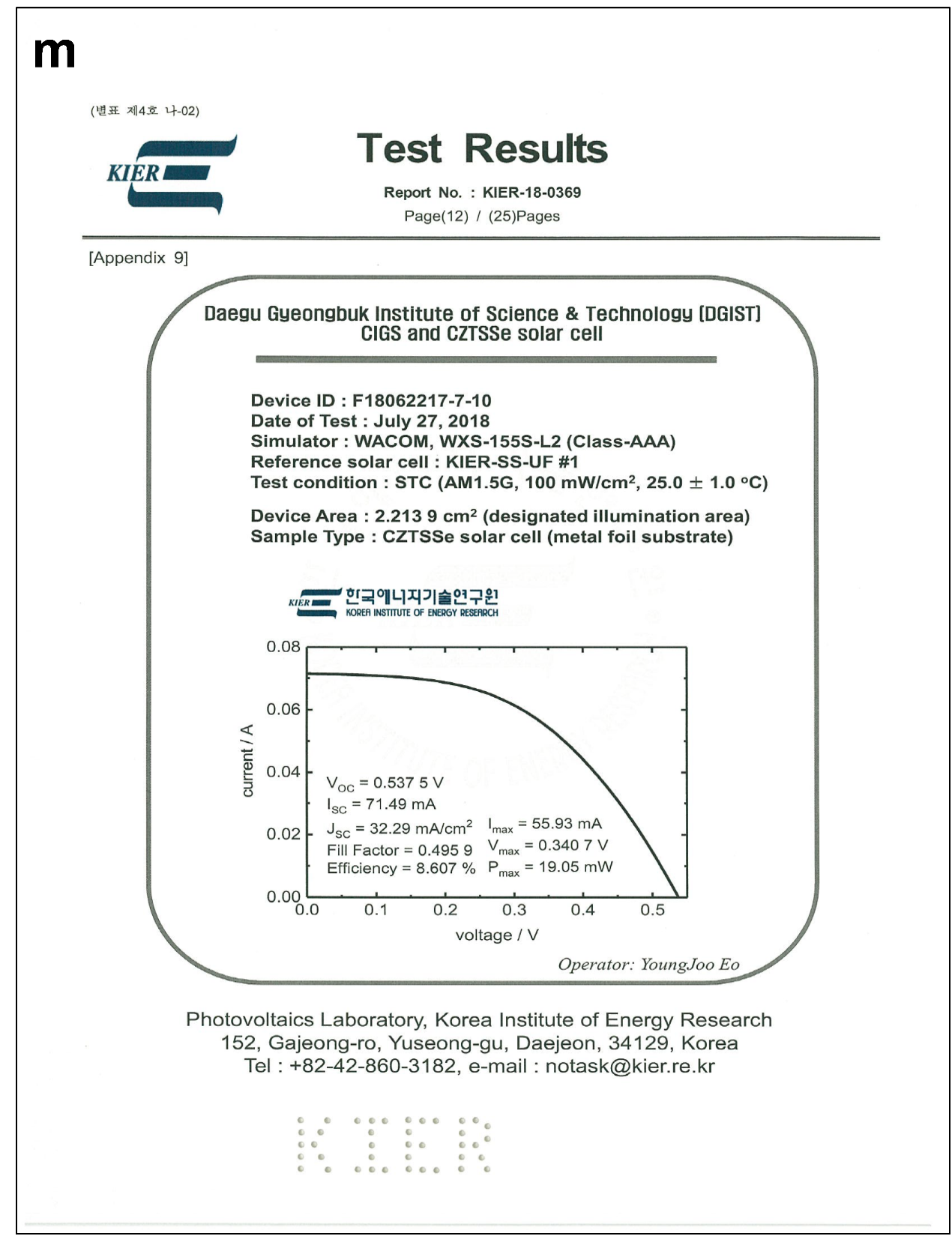


**Supplementary Figure. 8** *(continued)* **m** IV characteristics of an 8.607% efficiency cell with an area of 2.213 cm^2^.


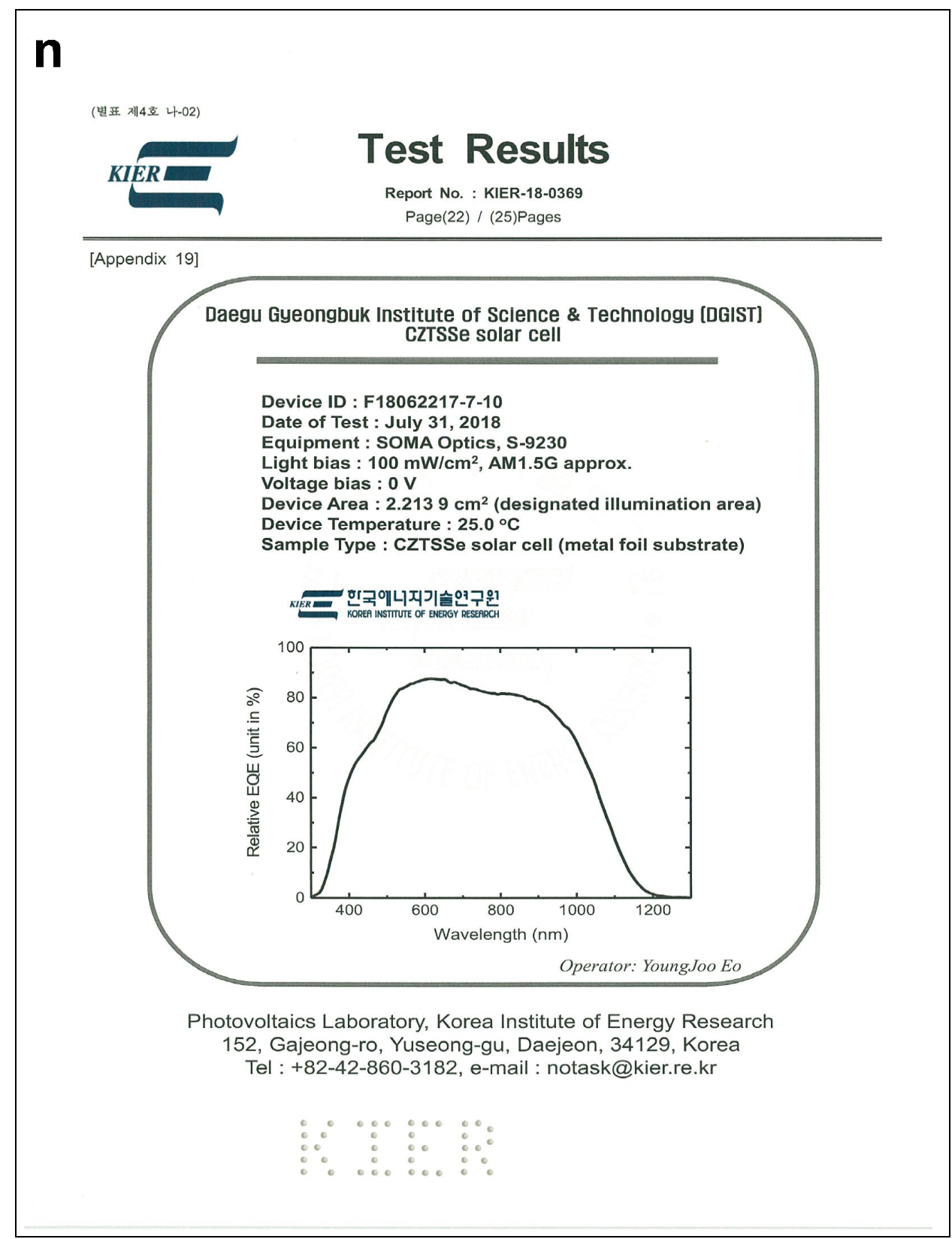


**Supplementary Figure. 8** *(continued)* **n** EQE characteristics of an 8.607% efficiency cell with an area of 2.213 cm^2^.


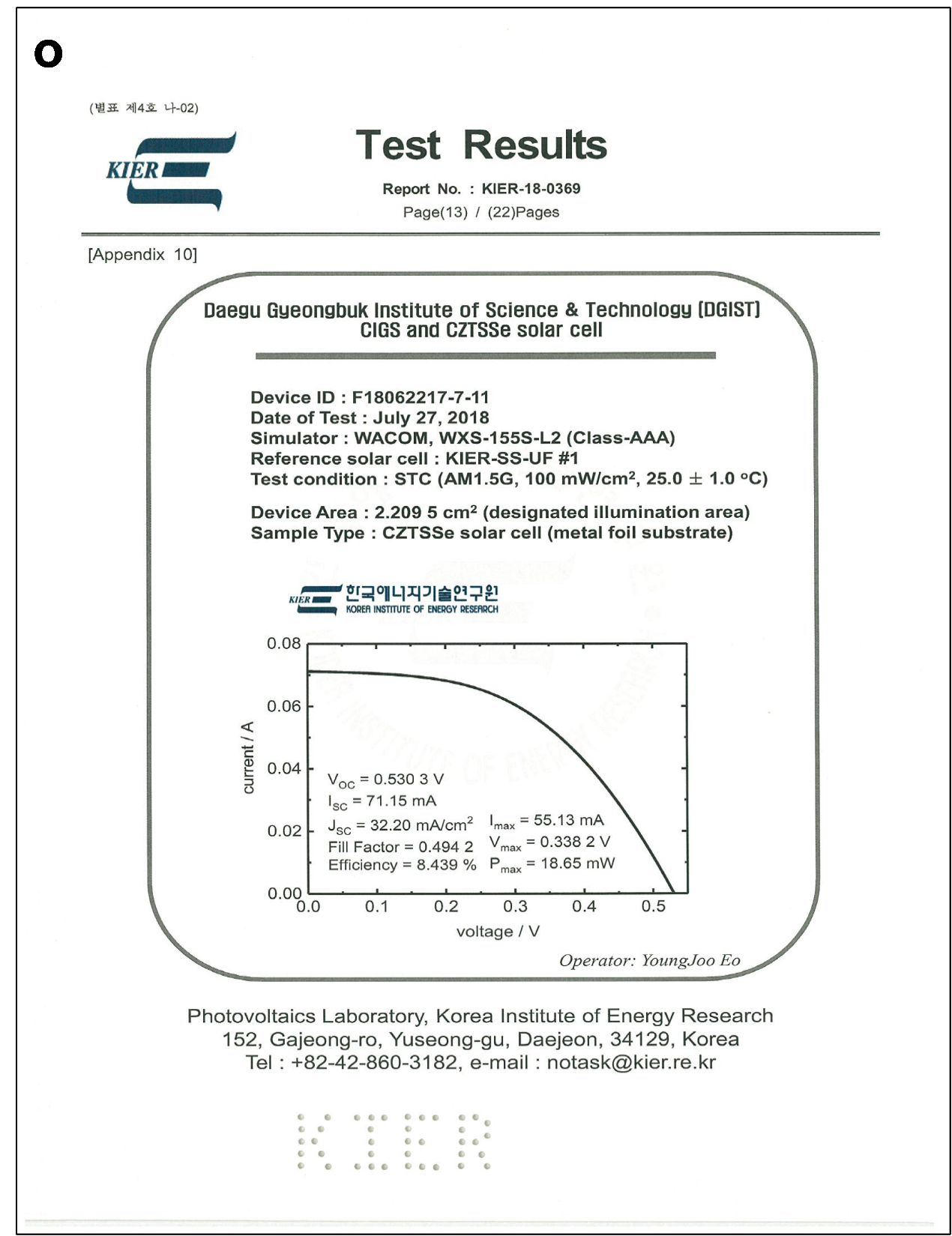


**Supplementary Figure. 8** *(continued)* **o** IV characteristics of an 8.439% efficiency cell with an area of 2.209 cm^2^.


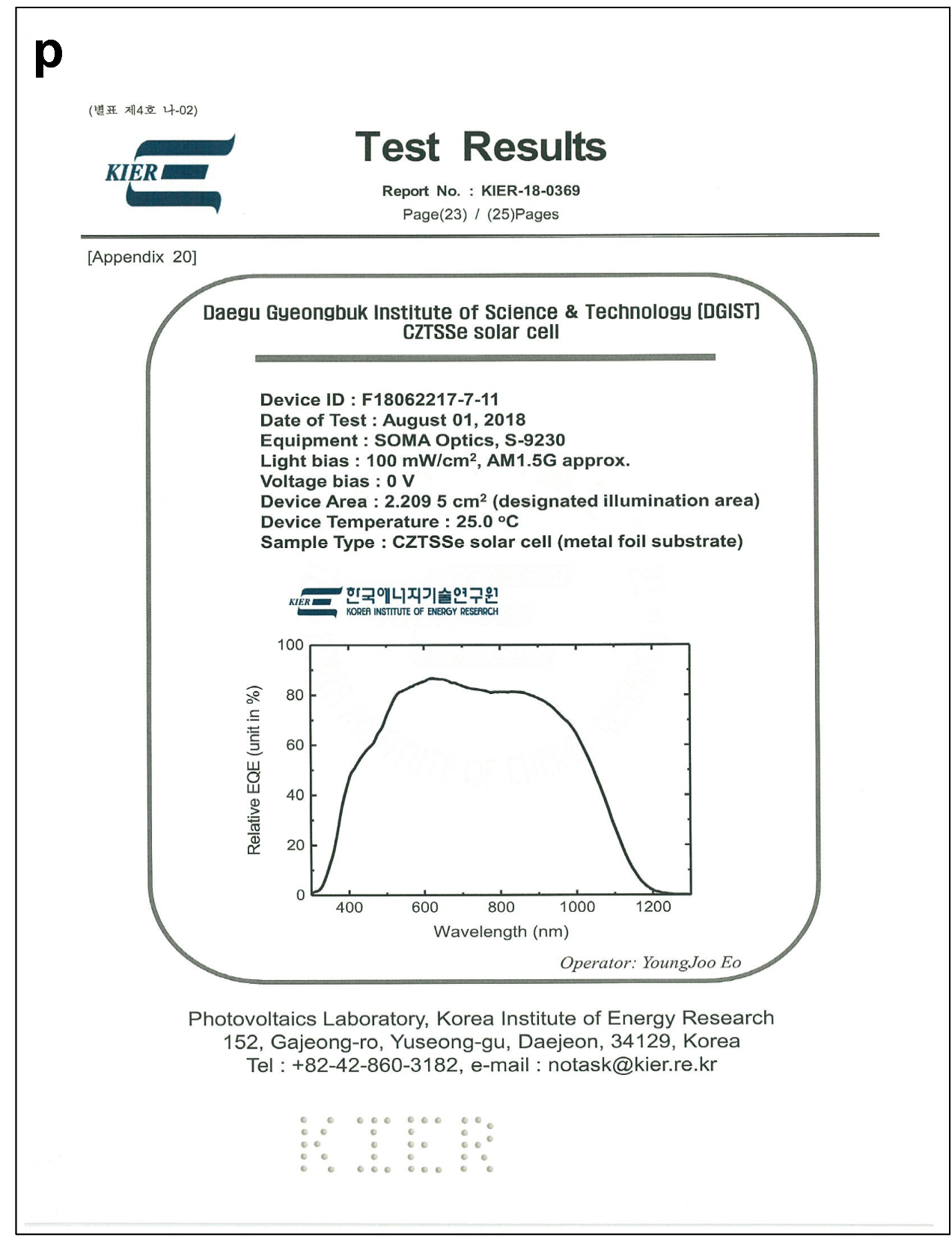


**Supplementary Figure. 8** *(continued)* **p** EQE characteristics of an 8.439% efficiency cell with an area of 2.209 cm^2^.


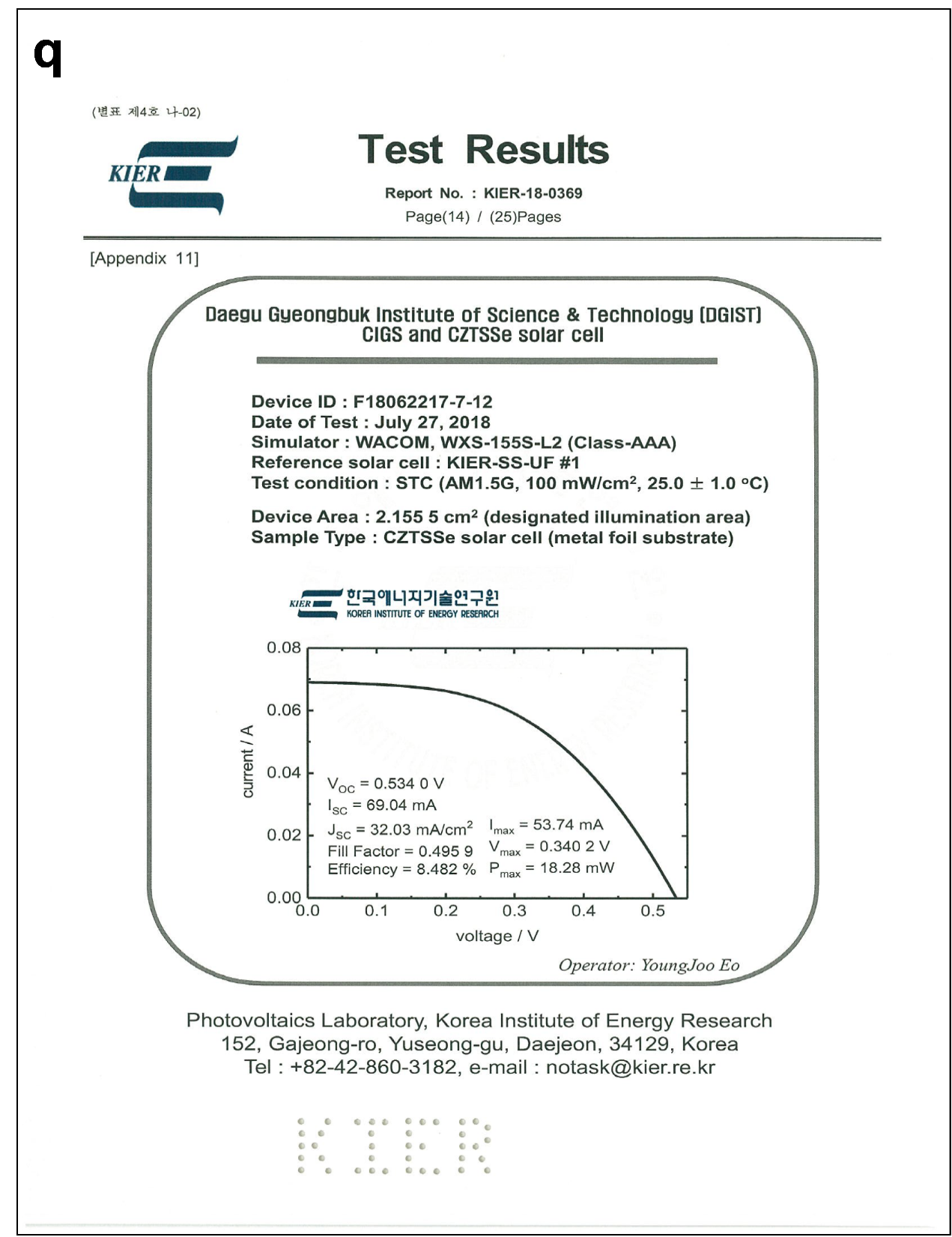


**Supplementary Figure. 8** *(continued)* **q** IV characteristics of an 8.482% efficiency cell with an area of 2.155 cm^2^.


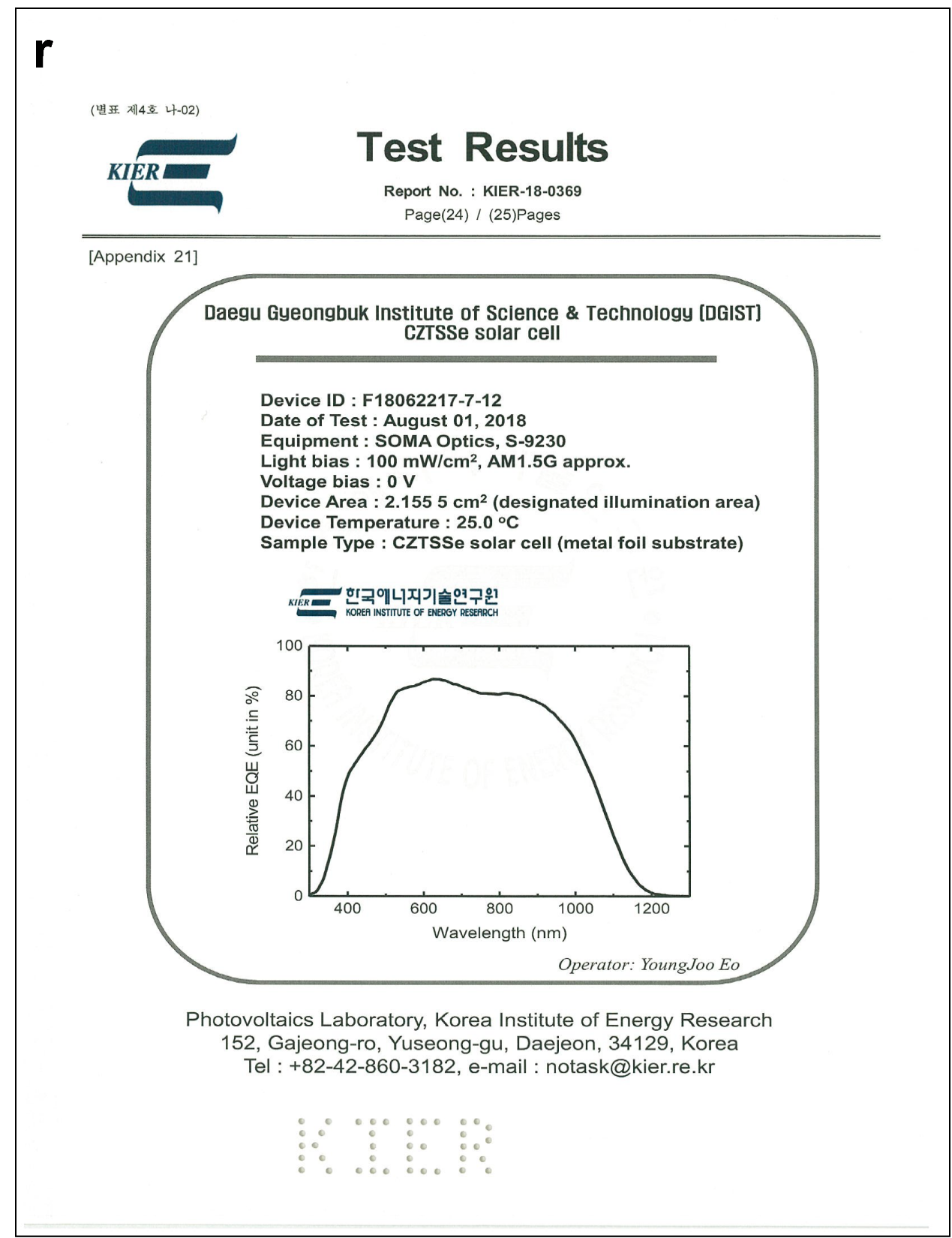


**Supplementary Figure. 8** *(continued)* **r** EQE characteristics of an 8.482% efficiency cell with an area of 2.155 cm^2^.

**References**

1. Walter, T., Herberholz, R., Müller, C. & Schock, H. Determination of defect distributions from admittance measurements and application to Cu(In,Ga)Se_2_ based heterojunctions. *J. Appl. Phys.* **80**, 4411–4420 (1996).
2. Igalson, M. & Zabierowski, P. Transient capacitance spectroscopy of defect levels in CIGS devices. *Thin Solid Films* **361–362**, 371–377 (2000).
