# Supplementary material for: Flexible Cu2ZnSn(S,Se)4 solar cells with over 10% efficiency and methods of enlarging the cell area
Source: Nat Commun. 2019 Jul 4;10:2959. doi: 10.1038/s41467-019-10890-x (PMC6609618; doi:10.1038/s41467-019-10890-x)
Supplement: Supplementary file 2 — Solar Cells Reporting Summary [file 41467_2019_10890_MOESM2_ESM.pdf]

nature research

Corresponding author(s): Kee-Jeong Yang, Jin-Kyu Kang

Solar Cells Reporting Summary

Nature Research wishes to improve the reproducibility of the work that we publish. This form is intended for publication with all accepted papers reporting the characterization of photovoltaic devices and provides structure for consistency and transparency in reporting. Some list items might not apply to an individual manuscript, but all fields must be completed for clarity.

For further information on Nature Research policies, including our data availability policy, see Authors & Referees.

Experimental design

Please check: are the following details reported in the manuscript?

1. Dimensions

Item	Yes/No	Details
Area of the tested solar cells	☒ Yes ☐ No	Area of the tested solar cells are described in Figs. 1, 3, Supplementary Figs. 1, 2, 4, 5, and the main text. Area of the tested small area solar cell labeled 'S' are active area (Supplementary Figs. 1 and 2). Area of the tested large area solar cell labeled 'L' are designated illumination area (Supplementary Figs. 4 and 5).
Method used to determine the device area	☒ Yes ☐ No	Active area is defined by scribing cell edge except grid area. Designated illumination area is defined by scribing cell edge. Area of the champion cells with small cell area and all cells with large cell area are reported and certified by KIER.

2. Current-voltage characterization

Item	Yes/No	Details
Current density-voltage (J-V) plots in both forward and backward direction	☐ Yes ☒ No	There is no reported influence of scan direction on CZTS-based thin-film solar cell J-V characteristics.
Voltage scan conditions<br>*For instance: scan direction, speed, dwell times*	☒ Yes ☐ No	Voltage scan conditions are described in “Methods” section of the main text.
Test environment<br>*For instance: characterization temperature, in air or in glove box*	☒ Yes ☐ No	Test environment is described in “Methods” section of the main text and Supplementary Figs .1, 2, 4, and 5.
Protocol for preconditioning of the device before its characterization	☒ Yes ☐ No	Protocol for preconditioning of the device before its characterization are described in “Methods” section of the main text.
Stability of the J-V characteristic<br>*Verified with time evolution of the maximum power point or with the photocurrent at maximum power point; see ref. 7 for details.*	☐ Yes ☒ No	No stability reported in this article.

3. Hysteresis or any other unusual behaviour

Item	Yes/No	Details
Description of the unusual behaviour observed during the characterization	☐ Yes ☒ No	There is no hysteresis and any other unusual behavior observed by authors and reported by KIER.
Related experimental data	☐ Yes ☒ No	There is no hysteresis and any other unusual behavior observed by authors and reported by KIER.

4. Efficiency

Item	Yes/No	Details
External quantum efficiency (EQE) or incident photons to current efficiency (IPCE)	☒ Yes ☐ No	EQE data are described in Figs. 1, 3, and Supplementary Figs. 1, 2, 4, and 5.
A comparison between the integrated response under the standard reference spectrum and the response measure under the simulator	☒ Yes ☐ No	The J_SC and EQE in Figs. 1e, f, and 3 are measured and certified by KIER (see Supplementary Figs. 1, 2, 4, and 5). The differences between the mention integrated responses were smaller than approximately 0.5% (varies depending test samples).
For tandem solar cells, the bias illumination and bias voltage used for each subcell	☐ Yes ☒ No	Solar cells in this article are not tandem solar cells.

5. Calibration

Item	Yes/No	Details
Light source and reference cell or sensor used for the characterization	☒ Yes ☐ No	It is described in “Methods” section of the main text.

Confirmation that the reference cell was calibrated and certified	☒ Yes ☐ No	It is described in "Methods" section of the main text.
Calculation of spectral mismatch between the reference cell and the devices under test	☒ Yes ☐ No	The spectral mismatches were between 0.995 – 1.005 depending on test samples. The spectral mismatch factor (M) was determined by KIER. Also, the champion cells of small area devices and all large area devices are certified by KIER.

6. Mask/aperture

Size of the mask/aperture used during testing	☐ Yes ☒ No	*Explain why this information is not reported/not relevant.*
Variation of the measured short-circuit current density with the mask/aperture area	☐ Yes ☒ No	Mask/aperture is not used.

7. Performance certification

Identity of the independent certification laboratory that confirmed the photovoltaic performance	☒ Yes ☐ No	For small area solar cell labeled 'S', the photovoltaic performance of 29 samples of CZTSSe3-S and those of CZZTSSe7-S are measured in DGIST. The photovoltaic performance of the champion cells with small cell area (Fig. 1e, f, Supplementary Figs. 1, 2) and that of all cells with large cell area (Fig. 3, Supplementary Figs. 4, 5) are reported and certified by KIER.
A copy of any certificate(s) *Provide in Supplementary Information*	☒ Yes ☐ No	Copies of certificates can be found in Supplementary Figs. 1, 2, 4, and 5.

8. Statistics

Number of solar cells tested	☒ Yes ☐ No	Number of CZTSSe3-S and CZTSSe7-S flexible solar cells with small cell area about 0.5 cm^2 are 30 devices, respectively (Fig. 1). Number of CZTSSe3-L and CZTSSe7-L flexible solar cells with large cell area over 2 cm^2 are 9 devices, respectively (Fig. 3).
Statistical analysis of the device performance	☒ Yes ☐ No	Statistical analysis is described in Figs. 1, 3, and the main text.

9. Long-term stability analysis

Type of analysis, bias conditions and environmental conditions *For instance: illumination type, temperature, atmosphere humidity, encapsulation method, preconditioning temperature*	☐ Yes ☒ No	No long-term stability analysis reported in this article.
